# Cleaved Delta like 1 intracellular domain regulates neural development via Notch signal-dependent and -independent pathways

**DOI:** 10.1242/dev.193664

**Published:** 2021-10-04

**Authors:** Yusuke Okubo, Fumiaki Ohtake, Katsuhide Igarashi, Yukuto Yasuhiko, Yoko Hirabayashi, Yumiko Saga, Jun Kanno

**Affiliations:** 1 Division of Cellular and Molecular Toxicology, Center for Biological Safety & Research, National Institute of Health Sciences, 1-18-1, Kamiyoga, Setagaya-ku, Tokyo 158-8501, Japan; 2 Institute for Advanced Life Sciences, Hoshi University, 2-4-41 Ebara, Shinagawa-ku, Tokyo 142-8501, Japan; 3 Life Science Tokyo Advanced Research center (L-StaR), Hoshi University School of Pharmacy and Pharmaceutical Science, 2-4-41 Ebara, Shinagawa-ku, Tokyo 142-8501, Japan; 4 Division of Mammalian Development, National Institute of Genetics, Yata 1111, Mishima 411-8540, Japan; 5 Department of Biological Science, Graduate School of Science, The University of Tokyo, Hongo 7-3-1, Bunkyo-ku, Tokyo 113-0033, Japan

**Keywords:** Delta-like 1 intracellular domain, Notch signaling, DRG development, Lateral inhibition, Map kinase pathway

## Abstract

Notch-Delta signaling regulates many developmental processes, including tissue homeostasis and maintenance of stem cells. Upon interaction of juxtaposed cells via Notch and Delta proteins, intracellular domains of both transmembrane proteins are cleaved and translocate to the nucleus. Notch intracellular domain activates target gene expression; however, the role of the Delta intracellular domain remains elusive. Here, we show the biological function of Delta like 1 intracellular domain (D1ICD) by modulating its production. We find that the sustained production of D1ICD abrogates cell proliferation but enhances neurogenesis in the developing dorsal root ganglia (DRG), whereas inhibition of D1ICD production promotes cell proliferation and gliogenesis. D1ICD acts as an integral component of lateral inhibition mechanism by inhibiting Notch activity. In addition, D1ICD promotes neurogenesis in a Notch signaling-independent manner. We show that D1ICD binds to Erk1/2 in neural crest stem cells and inhibits the phosphorylation of Erk1/2. In summary, our results indicate that D1ICD regulates DRG development by modulating not only Notch signaling but also the MAP kinase pathway.

## INTRODUCTION

Notch signaling regulates cell proliferation and cell fate decisions in dorsal root ganglia (DRG) development (Bhatt et al., 2013; Wakamatsu et al., 2000). During mouse development, by embryonic day 10.5 (E10.5) the migrating neural crest cells (NCCs) coalesce, giving rise to DRG in the trunk region (Marmigère and Ernfors, 2007). The NCCs differentiate to neuron and neural progenitor cells (NPCs), a process known as first-wave neurogenesis. After coalescence, the NPCs continue to proliferate, followed by differentiation to produce either neuron or glia, a process referred to as second-wave neurogenesis. Loss of Notch signaling in NCCs does not affect DRG formation and first-wave neurogenesis, but does prevent second-wave neurogenesis; the NPCs show precocious neuronal differentiation, resulting in reduced proliferation and increased apoptosis (Hu et al., 2011). Thus, loss of Notch signaling in the DRG leads to a decrease in cell number and to glial cell depletion. On the other hand, increased Notch signaling in NCCs results in an increase in cell proliferation and to inhibition of neuronal differentiation (Mead and Yutzey, 2012). Therefore, Notch signaling is required for optimal cell proliferation and gliogenesis during second-wave neurogenesis. In the developing mammalian nervous system, Notch, which is expressed on signal-receiving cells, is activated by expression of Delta-like 1 (Dll1) in neighboring signal-sending cells. Once activated, Notch signaling represses the expression of Dll1 by upregulating its downstream target Hes1, thereby making the receiving cells Dll1 negative. Thus, Dll1 and Hes1 show mutually exclusive expression patterns, a process known as lateral inhibition (Kageyama et al., 2008). During chick DRG development, it has been shown that the expression of *Delta1* mRNA also shows a ‘salt and pepper’ pattern among the neighboring cells. The proliferating cells in the developing nervous system subsequently undergo neuronal differentiation, thus gradually increasing the number of neurons. Later, the dividing cells give rise to satellite glial cells similar to mouse DRG. Based on these studies, it has been proposed that Notch signaling regulates proliferation and differentiation during DRG development via a lateral inhibition mechanism (Wakamatsu et al., 2000).

In mammals, there are five different Delta/Serrate/Lag2 (DSL) ligands [Delta-like 1 (Dll1), Dll3 and Dll4, and jagged 1 (Jag1) and Jag2] and four different Notch receptors. Upon binding with the DSL ligands (except Dll3) that are expressed on the surface of neighboring cells, the Notch receptor is first cleaved by a disintegrin and metalloprotease complex (ADAMs) followed by γ-secretase (Zolkiewska, 2008). The released Notch intracellular domain (NICD) translocates to the nucleus and activates transcription of Notch target genes such as *Hes* and *Hey* (Fischer and Gessler, 2007). It has been reported that Dll1, Jag1 and Jag2 are also cleaved by ADAMs and γ-secretase, and the intracellular domains are translocated to the nucleus (Ikeuchi and Sisodia, 2003; LaVoie and Selkoe, 2003). Few studies have shown the biological roles of the cleaved DSL intracellular domains (cDSL-ICDs) both *in vitro* and *in vivo*. D1ICD promotes neuronal differentiation in mouse neural stem cells (NSCs) by enhancing TGF-β signaling by binding to Smad2 and/or Smad3 proteins (Hiratochi et al., 2007). Jag1 intracellular domain regulates cardiac homeostasis in the mouse postnatal heart by inhibiting Notch signaling and activating Akt and Wnt signaling (Metrich et al., 2015). Moreover, D1ICD induces growth arrest in human umbilical vein endothelial cells (HUVECs) by upregulating the cell cycle inhibitor p21 (Kolev et al., 2005). Contrary to this, a few studies have reported that cDSL-ICDs have little effect on mouse embryogenesis and T-cell development (Liebler et al., 2012; Redeker et al., 2013). Therefore, the biological functions of cDSL-ICDs remain elusive.

In the present study, we generated two different genetically modified mice: one that overexpresses D1ICD using the Cre-loxP system and another that fails to produce D1ICD by deletion of genome sequences that are essential for the cleavage of Dll1. We show that, during DRG development, D1ICD overproduction promotes neurogenesis and inhibits proliferation, while inhibition of D1ICD production showed the opposite phenotype. With regards to the underlying molecular mechanism, we find that D1ICD acts as a component of the lateral inhibition by cooperating with Numb to repress Notch signaling, and that D1ICD also represses the MAP kinase pathway by inhibiting Erk1/2 phosphorylation.

## RESULTS

### D1ICD inhibits DRG cell proliferation

**Fig. 1.**
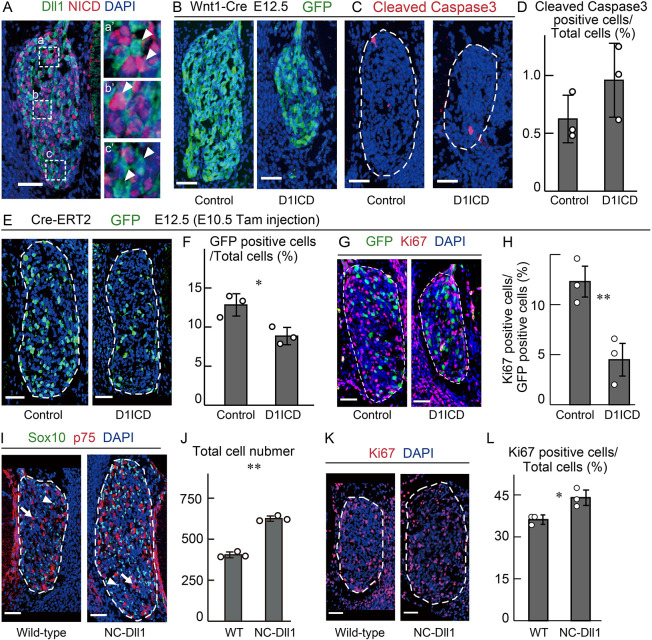
**D1ICD inhibits cell proliferation, resulting in cell number decreases in DRG during second-wave neurogenesis.** (A) Representative pictures showing immunostaining with anti-Dll1 (green) and anti-NICD (red) antibodies in the wild-type DRG at E12.5. Blue signals indicate nuclei. Each image on the right (a′, b′ and c′) shows the outlined area in a, b and c, respectively, at higher magnification. White arrowheads indicate Dll1-expressing cells. (B) Immunostaining showing GFP expression (green) in DRG sections derived from CAG-floxed CAT-GFP/Wnt1-cre (control) and CAG-floxed CAT-GFP/CAG-floxed-D1ICD/Wnt1-cre (D1ICD) embryos at E12.5. GFP expression indicates Cre-mediated recombination. (C,D) Immunostaining (C) and quantification (D) of apoptotic cells in DRG derived from control and D1ICD embryos, as shown in B. The ratio of apoptosis in DRG was calculated by counting three sections from one sample (*n*=3 different animals). (E,F) Immunostaining (E) and quantification (F) of the ratio of GFP-positive cells in DRG derived from CAG-floxed CAT-GFP/UBC-CreERT2 (control) and CAG-floxed CAT-GFP/CAG-floxed-D1ICD/UBC-CreERT2 (D1ICD) embryos at E12.5. Tamoxifen was injected at E10.5. The average number of GFP-positive cells from five DRG sections of three individuals are shown for control and D1ICD embryos (F). (G,H) Immunostaining (G) and quantification (H) of the ratio of Ki67-positive cells in DRG derived from control and D1ICD embryos using UBC-CreERT2 line (*n*=3 different animals). (I,J) Immunostaining (I) and quantification (J) of total cells in DRG derived from wild-type and NC-Dll1 embryos. The red and green colors represent p75 (arrows) and Sox10 (arrowheads), respectively. The average number of total DRG cells from three sections of three individuals are shown for control and NC-Dll1 embryos (J). (K,L) Immunostaining (K) and quantification (L) of the ratio of Ki67-positive cells in DRG derived from wild-type and NC-Dll1 embryos (*n*=3 different animals, all littermates). White dashed lines represent the DRG. Scale bars: 50 µm. Data are mean±s.d. Statistical analyses were performed using the two-tailed Student's *t*-test: *P<0.05, **P<0.01.

In order to analyze the role of D1ICD in DRG development, we first examined the expression pattern of Dll1 protein and Notch activity using specific antibodies. We focused on the trunk level DRG at E12.5, as it is well documented that the differentiation to neuron or glia from the common multipotent stem cells is regulated by Notch signaling at this stage (Hu et al., 2011). Dll1 expression and Notch activity showed roughly mutually exclusive patterns in the DRG at E12.5 (Fig. 1A). This result supports the idea that lateral inhibition via Notch-Delta signaling regulates DRG development in mice. To investigate whether D1ICD is involved in DRG development, we generated a transgenic mouse line carrying 3xHA_D1ICD_Flag under the control of the CAG promoter using the Cre-loxP system (Fig. S1A,B). NCC-specific expression of 3×HA_D1ICD_Flag was achieved by crossing the mice with a Wnt1-Cre mouse, which induces recombination in cranial, cardiac and trunk NCCs (Fig. 1B, left image; Fig. S1B) (Hu et al., 2011; Mead and Yutzey, 2012; Taylor et al., 2007; Yoshida et al., 2008). The recombination efficiency was confirmed by crossing the Wnt1-Cre mice with the CAG-CAT-GFP reporter mice; GFP was expressed in about 87.9±4.6% of the DRG cells at E12.5 (five sections were counted per animal, *n*=3 animals). Furthermore, we observed that D1ICD overproduction reduced the cell numbers and the DRG size at E12.5 (Fig. 1B, Fig. S2A). However, no difference in the ratio of apoptotic cells was observed in the D1ICD overproducing DRG compared with the control DRG (Fig. 1C,D). To exclude the possibility of migration defects of DRG precursors, D1ICD protein was induced by tamoxifen injection in UBC-CreERT2 and GFP-reporter mice from E10.5, when NCCs should have completed coalescence (Fig. S1B) (Ruzankina et al., 2007). We observed a decrease in the number of GFP-positive cells following sustained D1ICD production (Fig. 1E,F). Additionally, the expression of the cell proliferation marker Ki67 was decreased only in GFP-positive cells (Fig. 1G,H) and not in GFP-negative cells (Fig. S2B), indicating a reduction in the cell proliferation rate at E12.5. These results suggest that induced D1ICD inhibits cell proliferation after E10.5, when second-wave neurogenesis starts.

Next, we aimed to suppress the cleavage of endogenous Dll1. It has been reported that a specific 48 bp genome sequence is necessary for the production of D1ICD through successive cleavage by ADAM and γ-secretase (Fig. S1A) (Six et al., 2003). To confirm the suppression of D1ICD production, an expression vector containing the wild-type or non-cleavable Dll1 (NC-Dll1) was transfected into NIH3T3 cells expressing *Notch1* and *lunatic fringe* (*Lfng*), both of which are expressed in the migrating NCCs and enhances Notch1-Dll1 binding (Elena De Bellard et al., 2007; Taylor et al., 2014). In western blots using anti-Dll1 C-terminal antibody, D1ICD was detected in the wild-type Dll1 transfected cells, but not in NC-Dll1-transfected cells; even when an excess amount of protein was loaded, the NC-Dll1 transfected cell lysates did not show any bands, indicating that this mutant effectively produces non-cleavable Dll1 (Fig. S2C). We also evaluated the Notch signaling transduction ability of NC-Dll1 by co-culturing cells expressing NC-Dll1 with NIH3T3 cells expressing *Notch1*, *Lfng* and transfected TP1 luciferase Notch-reporter. We observed no difference in the Notch signaling transduction ability between the wild-type Dll1 and NC-Dll1 (Fig. S2D). In addition, to reproduce the Notch-Delta signal transduction found in native DRG development, we isolated neural crest stem cells (NCSCs) using FACS from E12.5 DRG obtained by crossing Wnt1-Cre mice with GFP-reporter mice. NCSCs can differentiate into neurons, glia and myofibroblasts upon withdrawal of treatment with growth factors (Nagoshi et al., 2008). Thus, we transfected Dll1 or NC-Dll1 into the NCSC, and then investigated the mRNA expression of Notch signaling target genes. The expression of *Hes1* and *Hey1* was not altered by the overexpression of Dll1 or NC-Dll1; however, there was a tendency towards a decrease in *Hes1* and an increase in *Hey1* expression levels (Fig. S2E). This result indicates that the function of NC-Dll1 as the Notch ligand is not very different from that of wild-type Dll1, even in NCSC. Based on these results, we generated a mouse line producing NC-Dll1 by deleting the 48 bp genome sequence through homologous recombination using the CRISPR-Cas9 system (Fig. S1A,C). We obtained two independent heterozygous F0 NC-Dll1 mice, which were indistinguishable from the wild-type mice. We then analyzed DRG development in the homozygous NC-Dll1 mice, which were viable and showed no obvious abnormalities except hyperactive behavior. Importantly, we observed contrasting phenotypes in the DRG at E12.5 between the NC-Dll1 mice and the D1ICD-expressing mouse. The DRG cell number and the cell proliferation rate, as measured by Ki67 immunostaining, were increased in the NC-Dll1 mice at E12.5 (Fig. 1I-L). Taken together, these results suggest that D1ICD negatively regulates cell proliferation in the DRG during the second wave neurogenesis.

### D1ICD enhances neuronal differentiation in DRG development

**Fig. 2.**
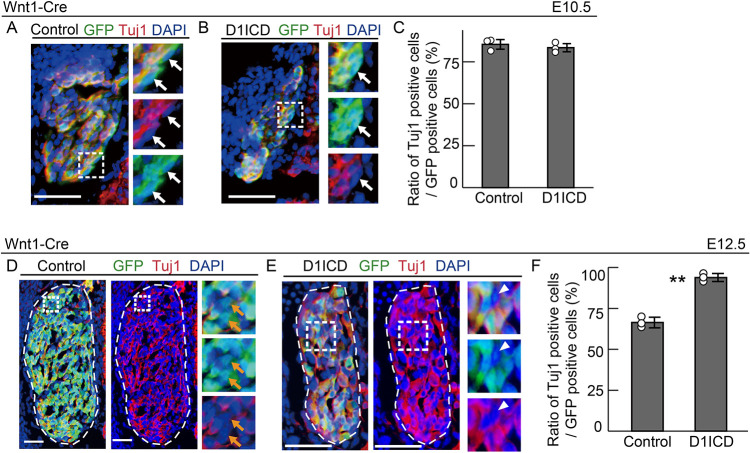
**D1ICD promotes neuronal differentiation at E12.5, but not at E10.5.** (A-C) Immunostaining showing expression of GFP (green) and Tuj1 (red) in DRG sections derived from CAG-floxed CAT-GFP/Wnt1-cre (A, control) and CAG-floxed CAT-GFP/CAG-floxed-D1ICD/Wnt1-cre (B, D1ICD) embryos at E10.5. Each image on the right shows the square outlined region at higher magnification. White arrows indicate Tuj1-negative cells in GFP-positive cells. Blue signal indicates nuclei. (C) The ratio of Tuj1-expressing cells to GFP-positive cells of control and D1ICD (*n*=3 different animals). (D-F) Immunostaining showing expression of GFP (green) and Tuj1 (red) in DRG sections using Wnt1-cre (D, control) and (E, D1ICD) embryos at E12.5. Orange arrows and white arrowheads represent Tuj1-negative cells in GFP-positive cells in control DRG and Tuj1-negative cells in GFP-negative cells in D1ICD-induced DRG, respectively. (F) The ratio of Tuj1-expressing cells among GFP-positive cells of control and D1ICD (*n*=3 different animals). Scale bars: 50 µm. Thin white dashed line represents DRG. Data are mean±s.d. Statistical analyses were performed using the two-tailed Student's *t*-test: **P<0.01.

**Fig. 3.**
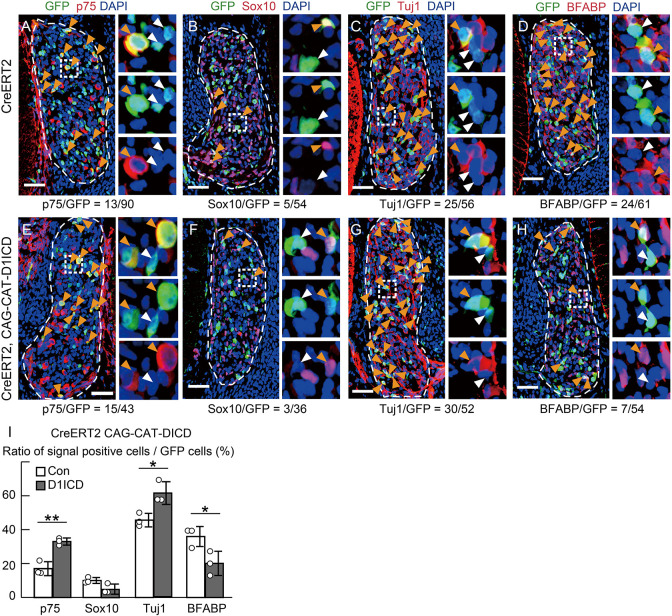
**D1ICD promotes neuronal differentiation and inhibits glial differentiation during second-wave neurogenesis.** (A-H) Immunostaining of DRG sections derived from CAG-floxed CAT-GFP/Cre-ERT2 (A-D, control) and CAG-floxed CAT-GFP/CAG-floxed-D1ICD/ Cre-ERT2 (E-H, D1ICD) embryos at E12.5. Tamoxifen was injected at E10.5. Each image on the right shows the square outlined region at higher magnification. Green indicates GFP. Red represents p75 (A,E), Sox10 (B,F), Tuj1 (C,G) and BFABP (D,H). The orange and white arrowheads represent marker-positive and -negative cells among GFP-positive cells, respectively. The numbers at the bottom in each picture represent the number of positive cells showing signal. (I) The ratio of marker-expressing cells to GFP-positive cells of control and D1ICD (*n*=3 different animals). Data are mean±s.d. Statistical analyses were performed using the two-tailed Student's *t*-test: *P<0.05, **P<0.01.

Overproduction of D1ICD in DRG resulted in deceased cell proliferation without affecting cell death, suggesting that D1ICD-expressing cells might have entered a premature differentiation pathway. To examine the effect of sustained D1ICD production in DRG neurogenesis, we performed immunostaining for several differentiation markers. During DRG development, the first wave neurogenesis occurs in migrating NCCs until E10.5 (Ma et al., 1999). It has been reported that Notch signaling acts only during second-wave neurogenesis after E10.5 (Hu et al., 2011; Taylor et al., 2007). Thus, we first examined the effect of D1ICD overproduction on the first wave neurogenesis by evaluating Tuj1 (neuron marker) expression in GFP (D1ICD)-positive cells induced by Wnt1-Cre at E10.5 (Fig. 2A,B). The ratio of Tuj1-positive cells among GFP-positive cells was not significantly different between the wild-type and D1ICD overproducing mice (Fig. 2C). Next, we examined the effect on second-wave neurogenesis at E12.5. Results showed that almost all the DRG cells producing D1ICD expressed Tuj1 (93.9±2.4%), which was significantly higher than those in the control DRG (66.4±3.2%) (Fig. 2D-F). To focus on second-wave neurogenesis more specifically, we induced D1ICD protein using UBC-CreERT2 and GFP-reporter mice after E10.5. Sustained D1ICD production promoted neurogenesis, increased the expression of neuronal precursor markers p75 and Tuj1, and decreased expression of the glial marker BFABP (Fig. 3). The expression of the glial precursor marker Sox10 was slightly decreased in the D1ICD-overexpressing cells; however, the decrease was not significant. These results suggest that D1ICD overproduction promotes neurogenesis and inhibits gliogenesis during second-wave neurogenesis.

**Fig. 4.**
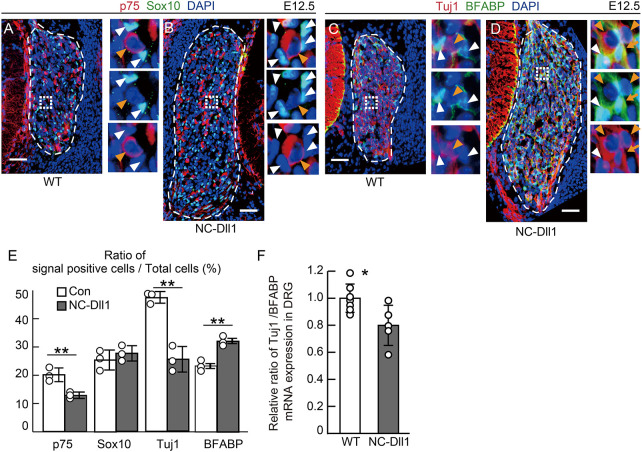
**Suppression of D1ICD production promotes glial differentiation and inhibits neuronal differentiation.** (A,B) Immunostaining showing expression of p75 (red) and Sox10 (green) of DRG sections derived from wild-type and NC-Dll1 embryos at E12.5. Blue signals indicate nuclei. (C,D) Immunostaining showing expression of Tuj1 (red) and BFABP (green) of DRG sections derived from wild-type and NC-Dll1 embryos at E12.5. Each image on the right shows the square outlined region at higher magnification. The orange and white arrowheads indicate P75 (A,B) or Tuj1 (C,D) and Sox10 (A,B) or BFABP (C,D), respectively. The orange arrows represent the cells co-expressing Tuj1 and BFABP. (E) The ratio of marker-expressing cells to total cells in wild-type and NC-Dll1 DRG at E12.5 (*n*=3 different animals, all littermates). Scale bars: 50 µm. The thin white dashed line outlines the DRG. (F) The relative ratio of Tuj1 to BFABP mRNA expression in DRG [*n*=7 (wild type), *n*=5 (NC-Dll1) different animals]. Data are mean±s.d. Statistical analyses were performed using the two-tailed Student's *t*-test: *P<0.05, **P<0.01.

Next, we investigated the function of endogenous D1ICD by inhibiting D1ICD production using NC-Dll1 homozygous mice at E12.5. We found that suppression of D1ICD production resulted in a decrease in the ratio of p75- and Tuj1-expressing cells, and an increase in the ratio of BFABP-expressing cells (Fig. 4A-E), which contrasts with the D1lCD overproduction phenotype. The ratio of Sox10-positive cells did not differ between the wild-type and NC-Dll1 DRG. In second-wave neurogenesis, common progenitors differentiate into neuronal or glial cells. The balance is regulated by Notch signaling via a lateral inhibition mechanism (Hu et al., 2011; Taylor et al., 2007; Wakamatsu et al., 2000). Thus, we examined the expression of marker genes in surgically isolated DRG of wild-type and NC-Dll1 mice at E12.5. The ratio of *Tuj1* mRNA expression per *Bfabp* mRNA expression decreased in the NC-Dll1 DRG, indicating that the repression of D1ICD production promoted cell differentiation toward glial cells (Fig. 4F). Taken together, these results suggest that D1ICD promotes neuronal differentiation and inhibits glial differentiation.

### D1ICD acts as an integral component of the lateral inhibition mechanism by repressing Notch activity

**Fig. 5.**
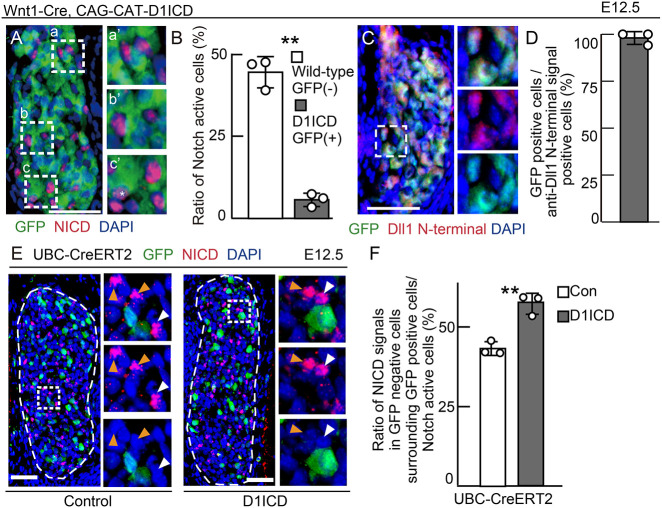
**D1ICD enhances and inhibits Notch activity in a non-cell autonomous and a cell-autonomous manner, respectively.** (A-D) Immunostaining of DRG sections derived from CAG-floxed CAT-GFP/CAG-floxed-D1ICD/Wnt1-cre embryos at E12.5. Each image on the right shows the square outlined region at higher magnification. Green indicates GFP (A,C). Red represent Notch activity (A) or Dll1 containing an N-terminal epitope (C). Each image on the right (a′, b′ and c′) shows the outlined area in a, b and c, respectively, at higher magnification. *n*=3 different animals. White asterisk indicates a Notch-active cell in GFP-positive cells (A). (B) The ratio of Notch-active cells to wild-type cells (white bar: GFP–negative) and D1ICD-induced cells (gray bar, GFP positive), respectively (*n*=3 different animals). (D) The ratio of GFP-positive cells to anti-Dll1 N-terminal signal-positive cells corresponding to C. *n*=3 (different animals). (E,F) Immunostaining (E) and quantification (F) of the ratio of NICD (red)-positive signals in GFP (green)-negative cells surrounding GFP-positive cells to Notch-active cell derived from CAG-floxed CAT-GFP/Cre-ERT2 (white bar) and CAG-floxed CAT-GFP/CAG-floxed-D1ICD/ Cre-ERT2 (gray bar) embryos at E12.5. Tamoxifen was injected at E10.5. White and orange arrowheads indicate active Notch signals in GFP-negative cells surrounding the GFP-positive cells and in GFP-negative cells, respectively (*n*=3 different animals). Scale bars: 50 µm. Thin white dashed lines represent DRG. Data are mean±s.d. Statistical analyses were performed using the two-tailed Student's *t*-test (B,F): **P<0.01.

As either the lack or overexpression of D1ICD was shown to influence second-wave neurogenesis in the DRG in which Notch signaling plays a crucial role, it can be hypothesized that D1ICD is involved in Notch signaling. In general, Notch signaling regulates neurogenesis via the lateral inhibition mechanism (Fig. S6A). A Delta-expressing neuronal precursor cell activates Notch signaling in the neighboring cells, leading to the induction of the downstream target genes *Hes* and *Hey*. The cells with active Notch signaling (Notch-active cells) proliferate or differentiate to glia. Delta expression is suppressed in the Notch-active cell; therefore, Notch signaling is suppressed in the cells adjacent to the Notch-active cells. As both Notch and Delta are membrane-bound proteins, the Notch- and Delta-mediated signaling usually requires close cellular proximity between adjacent cells; however, each adjacent cell is capable of sending the signal to the next. Therefore, the lateral inhibition regulates the cell number and differentiation in the whole DRG. To determine whether D1ICD participates in the Notch signaling pathway, we analyzed Notch activity-induced embryos produced by crossing the Wnt1-Cre with the CAG-CAT-D1ICD mice. We found that the NICD signal was absent in most of the D1ICD-induced cells but was strongly observed in the GFP-negative neighboring cells (Fig. 5A,B). Moreover, the D1ICD overproduction enhanced BFABP expression in the neighboring cells (Fig. S3A,B). Next, we looked for endogenous Dll1 protein expression under the control of Notch signaling by immunostaining using an anti-Dll1 N-terminal region antibody. Endogenous Dll1 signal was detected only in the GFP-positive cells, indicating that endogenous Dll1 expression was repressed in the Notch-active neighboring cells via the lateral inhibition mechanism (Fig. 5C,D). Next, we attempted to induce D1ICD expression sparsely by tamoxifen injection in the UBC-CreERT2/CAG-CAT-D1ICD mice, and analyzed its role in neighboring cells. Sparse expression of D1ICD had no significant effect on the overall ratio of total NICD-positive cells in the DRG compared with the control (Fig. S3C). However, Notch-active cells were more frequently found near D1ICD-overproducing cells than around the control cells expressing only GFP (Fig. 5E,F). This result suggests that D1ICD overproduction increases Notch activity in the adjacent cells. Thus, D1ICD-expressing cells might repress their own Notch activity.

**Fig. 6.**
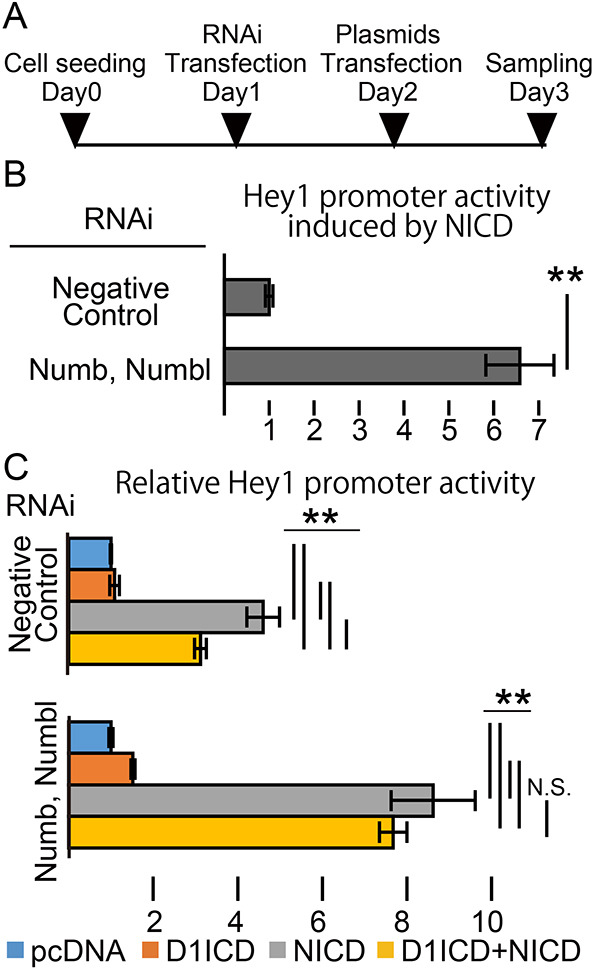
**D1ICD inhibits Notch activity in coordination with the Notch signal inhibitor Numb.** (A) The experimental procedure for the Hey1 promoter assay. (B,C) Hey1 promoter assay transfected with the Luciferase expression vector under the control of Hey1 promoter activity. (B) Hey1 promoter activity induced by NICD in the condition of Numb and Numbl knockdown. (C) Hey1 promoter activity transfected with NICD and/or D1ICD under the Numb and Numbl knockdown condition (*n*=3 independent transfection experiments). Data are mean±s.d. Statistical analyses were performed using the two-tailed Student's *t*-test (B) and the one-way ANOVA with Tukey's post-hoc tests for multiple comparisons (C): **P<0.01.

It has been reported that, during cell division, the asymmetric distribution of Numb protein, which works as a Notch signaling inhibitor (Chapman et al., 2006; McGill and McGlade, 2003), regulates cell fate decisions in the developing chick DRG (Wakamatsu et al., 2000), and Numb inhibits NICD nuclear localization in isolated mouse DRG cells (Huang et al., 2005). Thus, we speculated that Numb is involved in the D1ICD-mediated suppression of Notch activity. To test this possibility, we performed Hey1 promoter assays in NIH3T3 cells transfected with D1ICD or NICD, or both, under the Numb knockdown condition (both Numb and Numbl were deleted because Numbl is known to compensate for Numb function; Huang et al., 2005). *Hey1*, a Notch signaling target gene, suppresses neuronal differentiation in a subpopulation of DRG sensory neurons (Mukhopadhyay et al., 2009) and in the neural progenitor cells in the brain (Sakamoto et al., 2003). We used siRNA to silence Numb and Numbl expression and examined its efficiency using western blotting. Numb and Numbl protein expression disappeared 48 h after of siRNA transfection (Fig. S4A-C). We transfected the siRNAs and Hey1-reporter plasmid set (Luciferase expression vector under the control of Hey1 promoter, NICD and/or D1ICD expression plasmids) into the cells at 24 h and 48 h after cell seeding, respectively (Fig. 6A). 72 h after cell seeding, Hey1 promoter activity induced by NICD was significantly increased in the absence of Numb and Numbl (Fig. 6B). This result confirmed previous reports that Numb and Numbl repress Notch signaling (Huang et al., 2005; Zilian et al., 2001). Next, we also investigated the role of D1ICD against Notch signaling. Results showed that D1ICD itself did not change Hey1 promoter activity; however, D1ICD inhibited the Hey1 promoter activity induced by NICD (Fig. 6C, upper graph). The result suggests that D1ICD inhibits Notch signal in a cell-autonomous manner. Next, we asked whether D1ICD repressed Notch activity even in the double-knockdown condition. D1ICD-mediated reduction in the Hey1 promoter activity was canceled in the Numb/Numbl double-knockdown condition (Fig. 6C, bottom graph). These results indicate the possibility that D1ICD inhibits Notch signaling in cooperation with Numb and Numbl.

**Fig. 7.**
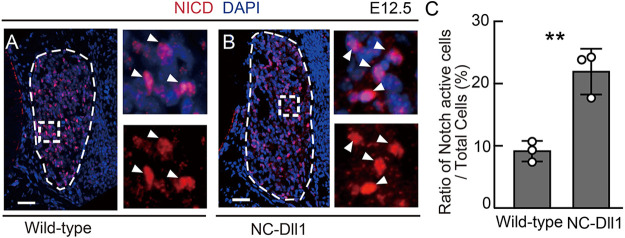
**The repression of D1ICD production increases Notch signal activity.** (A-C) Immunostaining (A,B) and quantification (C) of anti-NICD (red) in wild-type (A) and NC-Dll1 DRG (B) at E12.5. Each image on the right shows the square outlined region at higher magnification. White arrowheads indicate cells with active Notch signal. Blue signals indicate nuclei (*n*=3 different animals, all littermates). (C) The ratio of cells with active Notch signal to total cells of wild-type and NC-Dll1 DRG at E12.5. Scale bars: 50 µm. Thin white dashed lines represent DRG. Data are mean±s.d. Statistical analyses were performed using the two-tailed Student's *t*-test (B): **P<0.01.

To further investigate the possible role of D1ICD, we examined the Notch activity in NC-Dll1 DRG that lacks D1ICD production. We found that the ratio of Notch active cells was increased in NC-Dll1 DRG (Fig. 7). Notch activity is regulated via a lateral inhibition mechanism. In our experiment, Notch activity and Dll1 expression showed a roughly mutually exclusive pattern in the wild-type DRG at E12.5 (Fig. 1A). Therefore, we explored whether the upregulation of Notch signaling in NC-Dll1 DRG was caused by the disruption of the lateral inhibition mechanism. We found that Notch activity and Dll1 expression showed a salt-and-pepper pattern in NC-Dll1, as shown in the wild type, and the ratio of cells co-stained with the NICD and Dll1 C-terminal epitope in NICD-positive cells did not change significantly between the wild-type and NC-Dll1 DRGs (Fig. S4D,E). These results indicate that the lateral inhibition mechanism also worked properly even in NC-Dll1, and the upregulation of Notch activity is because of a lack of Notch activity modulation by D1ICD rather than disruption of the lateral inhibition mechanism. Taken together, these results suggest that D1ICD functions as a component of the lateral inhibition mechanism by repressing Notch signaling in the same cell.

### D1ICD enhances neuronal differentiation in a Notch-independent manner

**Fig. 8.**
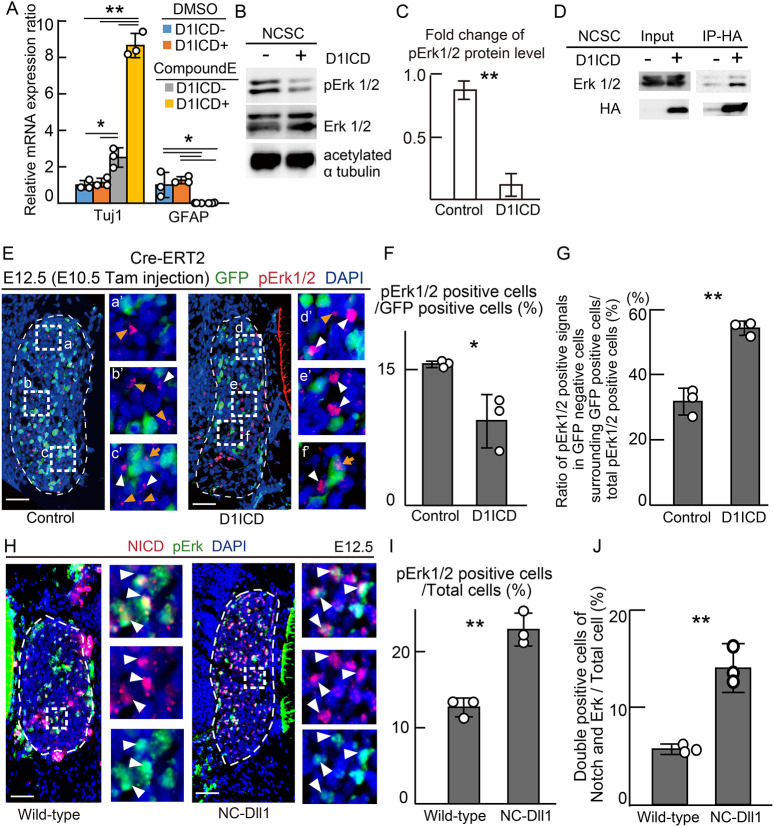
**D1ICD inhibits and enhances Erk1/2 phosphorylation in a cell-autonomous and non-cell-autonomous manner, respectively.** (A) The relative mRNA expression ratio in NCSCs derived from CAG-floxed CAT-GFP/Wnt1-cre (blue and gray bars) and CAG-floxed CAT-GFP/CAG-floxed-D1ICD/Wnt1-cre (orange and yellow bars) DRGs after treatment with the γ-secretase inhibitor compound E (*n*=3 different embryo pool sets). The relative GFAP expression was 0.005±0.002 and 0.011±0.016 in D1ICD^−^ and D1ICD^+^ NCSC treated with compound E, respectively. (B,C) Protein expression (B) and quantification (C) of phosphorylated Erk1/2, Erk1/2 and acetylated α-tubulin (internal control) in each NCSC. (C) The graph represents the quantitative analysis of phosphorylated Erk1/2 protein levels and the data normalized to total Erk1/2 levels using the same cell lysates (*n*=3 from different embryo pool sets). (D) Immunoprecipitation experiments of 3×HA_D1ICD_Flag in each NCSC using an anti-HA antibody. Erk1/2 and HA proteins were detected using western blotting. (E-G) Immunostaining (E) and quantification (F,G) of sections derived from CAG-floxed CAT-GFP/Cre-ERT2 (control) and CAG-floxed CAT-GFP/CAG-floxed-D1ICD/ Cre-ERT2 (D1ICD) embryos at E12.5. Tamoxifen was injected at E10.5. Each image on the right (a′, b′, c′, d′, e′ and f′) shows the square outlined areas at higher magnification (a, b, c, d, e and f). Blue signals indicate the nuclei. The white and orange arrowheads indicate phosphorylated Erk1/2 signals in GFP-negative cells surrounding GFP-positive and GFP-negative cells, respectively. The orange arrows represent phosphorylated Erk1/2 signals in GFP-positive cells. (F) The ratio of pErk1/2-positive cells to GFP-positive cells. (G) The ratio of pErk1/2 positive signals to GFP-negative cells surrounding GFP-positive cells per total pErk1/2-positive cells (*n*=3 different animals). (H-J) Immunostaining (H) and quantification (I,J) of sections derived from wild-type and NC-Dll1 DRG. Each image on the right shows the outlined region at higher magnification. White and orange arrowheads indicate co-stained cells and stained cells, respectively, using Notch activity and phosphorylated Erk1/2 antibodies. (I) The ratio of pErk1/2-positive cells among the total cells of the DRG in wild-type and NC-Dll1. (J) The ratio of cells double-positive for Notch signaling and the MAP kinase pathway to total cells of the DRG in wild-type and NC-Dll1 (*n*=3 different animals and littermates). Scale bars: 50 µm. The thin white dashed lines represent the DRG. Data are mean±s.d. Statistical analyses were performed using the two-tailed Student's *t*-test (C,F,G,I,J) and one-way ANOVA with Tukey's post-hoc tests for multiple comparisons (A): *P<0.05, **P<0.01.

Next, we investigated a hypothesis that D1ICD could promote neuronal differentiation in a Notch signaling-independent manner because it is reported that D1ICD promotes neuronal differentiation in NSCs by activating TGF-β/activin signaling through binding to Smad proteins (Hiratochi et al., 2007). To examine this possibility, we used NCSCs expressing D1ICD isolated from the DRG of E12.5 embryos by crossing of CAG-CAT-D1ICD mice with Wnt1-Cre and GFP-reporter mice. NCSCs expressing GFP were sorted by FACS using an anti-GFP antibody. To confirm the D1ICD function, NCSCs were differentiated for 5 days in the absence of growth factors. *Tuj1* mRNA expression was increased in D1ICD-expressing NCSCs compared with control NCSCs, although the expression of glial marker *Gfap* and myofibroblast marker α-smooth muscle actin (αSMA; *Acta2*) was not different (Fig. S5A). These results suggest that D1ICD promotes neurogenesis in NCSCs. Next, we conducted the differentiation assay using exactly the same NCSC population as the material by withdrawing growth factors and treating with the γ-secretase inhibitor compound E, to elucidate the possible function of D1ICD independent of Notch signaling. We confirmed that Notch signaling was inhibited by compound E treatment, because *Hes1* mRNA expression was decreased (Fig. S5B). D1ICD significantly increased *Tuj1* expression even in the NCSCs treated with compound E (Fig. 8A), although under these experimental conditions, we did not observe upregulation of *Tuj1* alone by D1ICD (see Discussion). These results indicate that D1ICD promotes neuronal differentiation independently of endogenous Notch signaling.

### D1ICD inhibits MAP kinase pathway in NCSCs

Next, we investigated the molecular mechanism of D1ICD-mediated neuronal differentiation in a Notch signaling-independent manner. The vertebrate D1ICD protein contains a nuclear localization signal and a PDZ-binding motif, but no typical DNA-binding motifs (Hiratochi et al., 2007). Thus, we speculated that D1ICD partners with other DNA-binding proteins in the nucleus. It had been reported that D1ICD subcellular localization was different in different cell types; nuclear D1ICD was detected in mouse NSCs, HEK293T cells and HUVECs (Hiratochi et al., 2007; Jung et al., 2011; Kolev et al., 2005; Liebler et al., 2012; Six et al., 2003), but not in CHO cells (Redeker et al., 2013). Moreover, nuclear D1ICD is degraded rapidly by the action of proteases (Dyczynska et al., 2007; Six et al., 2003). We first confirmed the nuclear localization of D1ICD and further showed D1ICD accumulation following treatment with the protease inhibitor epoxomicin in NCSCs (Fig. S5C,D). To determine the possible binding partners of D1ICD, we performed immunoprecipitation using anti-HA and anti-Flag antibodies in NCSCs overexpressing the D1ICD, followed by mass spectrometry (IP-MS/MS). As the amount of D1ICD protein in the cells was too low, we could not detect any protein, including D1ICD, by MS. Thus, we used HEK293T cells transfected with 3×Flag_D1ICD, and precipitated and detected the D1ICD-binding proteins by IP-MS/MS using the anti-Flag antibody (Tables S1 and S2). The immunoprecipitated proteins were functionally annotated using the Database of Annotation, Visualization, and Integrated Discovery (DAVID) (Table 1). To confirm successful precipitation of the bait, we also searched mouse database, and identified peptides corresponding to mouse D1ICD (Table S3). We detected membrane-associated guanylate kinase, WW and PDZ domain-containing protein (MAGI) 1 and MAGI3 proteins that are known D1ICD-binding proteins (Mizuhara et al., 2005; Wright et al., 2004), indicating that our analysis was reliable. The IP-MS/MS experiment was performed twice and we found that the cell cycle regulators [cyclin dependent kinase (CDK) 1, CDK2, and CDK4] and mitogen-activated protein (MAP) kinase signaling pathway members [extracellular signal-regulated-kinase (ERK) 1, ERK2, mitogen-activated protein kinase 14 (MAPK14) and growth factor receptor-bound protein 2(GRB2)] are the possible binding partners in both the experiments. It has been reported that the inhibition of ERK1 and/or ERK2 phosphorylation promotes neuronal differentiation and suppresses proliferation in NSCs (Wang et al., 2009). Furthermore, CDK2 and cyclin D1 protein levels were downregulated by the inhibition of ERK1 and/or ERK2 phosphorylation (Wang et al., 2009). Therefore, we hypothesized that ERK1, ERK2 or CDKs, or all three, regulate neuronal differentiation and cell proliferation via D1ICD. To investigate these possibilities, we first examined the expression of these proteins in D1ICD-expressing NCSCs by following growth factor withdrawal using western blotting. The expression levels of CDK2 and CDK4 were not altered during the 5 days of culture in differentiation-inducing conditions (Fig. S5E). In addition, the protein levels of ERK1 and ERK2 did not change, but the level of phosphorylated ERK1 and ERK2 (pErk1/2) was decreased (Fig. 8B,C). We also confirmed the D1ICD-ERK1 and -ERK2 interaction in NCSCs by immunoprecipitation and western blotting (Fig. 8D). These results indicate that D1ICD represses ERK1 and ERK2 phosphorylation in NCSCs.

**Table 1.**
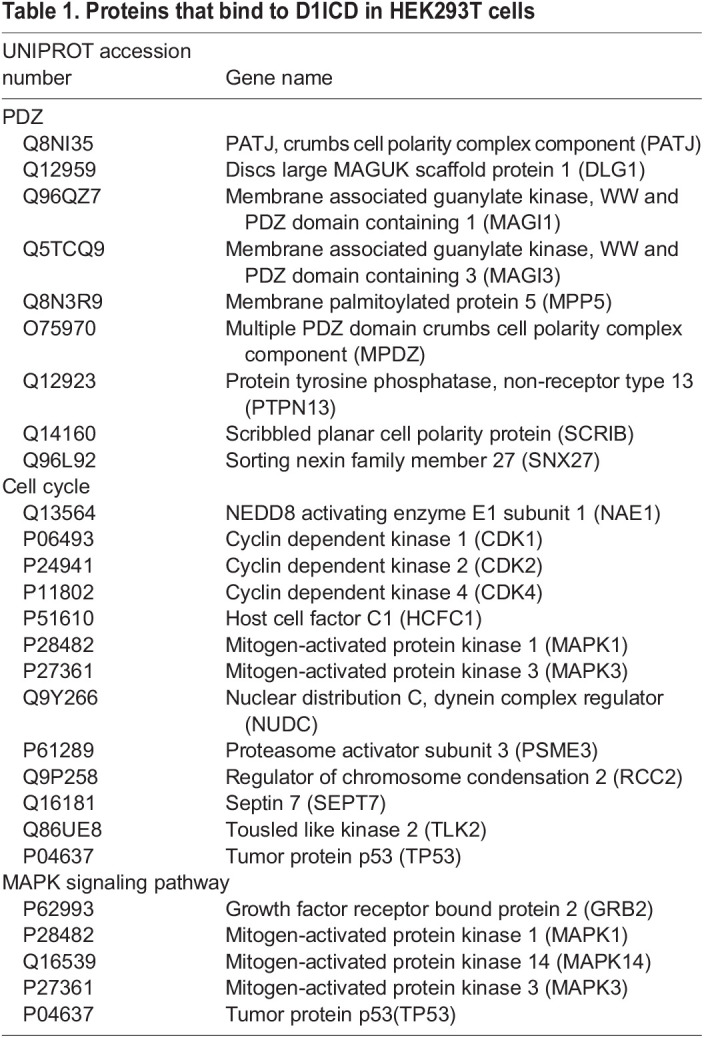
Proteins that bind to D1ICD in HEK293T cells

### D1ICD suppressed phosphorylation of ERK1 and ERK2

To further investigate the role of D1ICD in regulating ERK1 and ERK2 activity *in vivo*, we performed immunostaining for pErk1/2 at E12.5. We found that the ratio of pErk1/2-positive cells among GFP-positive cells was decreased in the E12.5 DRGs overproducing D1ICD from E10.5 using UBC-CreERT2 (Fig. 8E,F). In contrast, the ratio of pErk1/2-positive cells was increased in the NC-Dll1 DRG compared with the wild-type DRG (Fig. 8H,I). These results indicate that D1ICD binds to ERK1 and ERK2 and inhibits their phosphorylation during the second wave neurogenesis. As described above, D1ICD represses Notch signaling in the same cells and activates Notch signaling in the neighboring cells (Fig. 5A,B,E,F). Thus, we investigated whether D1ICD overproduction affects the MAP kinase pathway in the adjacent cells. The ratio of pErk1/2-positive cells was significantly increased in D1ICD-negative cells surrounding D1ICD-positive cells (Fig. 8G). Moreover, we found that the ratio of double-positive cells of NICD and phosphorylated ERK1 and ERK2 was increased in the NC-Dll1 DRG (Fig. 8J). Notch signal was enhanced in the NC-Dll1 DRG compared with the wild-type DRG (Fig. 7). These results indicate that Notch signal and Map kinase pathways are coordinately regulated in second-wave neurogenesis.

## DISCUSSION

In this study, we assessed the biological roles of D1ICD in DRG development. Our results lead to several clear conclusions. First, D1ICD inhibits cell proliferation and promotes neuronal differentiation during second-wave neurogenesis. Second, D1ICD functions as an integral component of the lateral inhibition mechanism by suppressing Notch signaling. Third, D1ICD represses Notch activity in coordination with Numb in NIH3T3 cells. Finally, D1ICD binds ERK1 and ERK2 and inhibits their phosphorylation in a cell-autonomous manner. Taken together, our findings indicate the functional significance of D1ICD in DRG development.

To investigate the possible role of D1ICD, Redeker et al. (2013) reported the establishment of a knock-in mouse line in the HPRT locus, in which D1ICD could be expressed ubiquitously under the control of a CAG promoter (Redeker et al., 2013). In these mice, the expression level of Neurog1 was similar to that in the wild-type mice at E9.5, as assessed by whole-mount *in situ* hybridization. Moreover, the mRNA expression levels of both a pan-neuronal marker, Nefm, and a neuronal marker, Islet1, in the E9.5 embryos of the mice was higher when compared with the wild-type embryos, although the difference was not statistically significant. The discrepancy between these studies and ours might be due to the differences in the developmental stages and tissues analyzed as we also did not observe any difference up to E10.5 before the second wave neurogenesis in DRG.

Notch signaling regulates central and peripheral nervous system development by controlling cell proliferation and differentiation via a lateral inhibition mechanism. In a previously established model (Fig. S6A), Notch signaling promoted cell proliferation and glial differentiation by repressing proneural genes and their downstream target Dll1. On the other hand, in the neighboring Notch-inactive cells, the expression of proneural genes and Dll1 was upregulated, resulting in neuronal differentiation. This balance is tuned by Notch signaling via lateral inhibition mechanism among the DRG cells (Kageyama et al., 2008; Wakamatsu et al., 2000). Moreover, Numb protein showed asymmetrical inheritance into one daughter cell. Thus, Notch signaling is inhibited in the Numb-inherited cell, leading to its differentiation into neuronal cells. Here, we modified the lateral inhibition model by including the D1ICD function (Fig. S6B). D1ICD inhibits Notch signaling in coordination with Numb. As a result, the expression of proneural genes is elevated to promote neuronal differentiation, together with the upregulation of Dll1. Therefore, the increased Dll1 strongly activates Notch signaling in the neighboring cells. Furthermore, our study reveals that D1ICD promotes neuronal differentiation in NCSCs in a Notch signal-independent manner (Fig. 8A). As shown in Fig. S5A, we found that D1ICD-overexpressing NCSCs significantly increased Tuj1 in the differentiation assay upon withdrawal of growth factors. In contrast, D1ICD did not increase Tuj1 expression (Fig. 8A), although we used the same NCSC population. The only difference was the absence (Fig. S5A) or presence (Fig. 8A) of DMSO. It has been reported that the mRNA expression of the neuronal marker doublecortin decreased in adult rat neural stem and precursor cells treated with 1% DMSO, indicating that low concentrations of DMSO suppress neuronal differentiation (O'Sullivan et al., 2019). Therefore, we speculated that low concentrations of DMSO also inhibited neuronal differentiation in the NCSCs. Nevertheless, based on the strong upregulation of Tuj1 by D1ICD even in the presence of compound E, we conclude that D1ICD promotes neuronal differentiation even when endogenous Notch signaling is repressed. Moreover, D1ICD inhibited the phosphorylation of ERK1 and ERK2 during the second wave neurogenesis (Fig. 8B-I). ERK1 and ERK2 bind to D1ICD in NCSC, suggesting that the MAP kinase pathway is a direct target of D1ICD. ERK1 and ERK2 function as effectors of ErbB signaling and play an important role in the survival of DRG-derived glial cells (Newbern et al., 2011). We found that D1ICD binds not only ERK1 and ERK2 but also Grb2, which is required for ERK1 and ERK2 activation mediated by ErbB signaling (Fig. 8D and Table 1) (Mei and Nave, 2014). These results indicate the possibility that D1ICD also regulates gliogenesis in coordination with ErbB signaling by inhibiting the ERK1 and ERK2 phosphorylation. However, we did not elucidate the direct interaction of D1ICD and ERK1 and ERK2 in DRG; thus, the results might indicate an indirect consequence of changes in cell proliferation or differentiation. Further analysis of the DICD and MAP kinase pathways should be an important subject for future studies.

In this study, although we could not address the role of endogenous D1ICD in Dll1 function, which activates Notch signaling in neighboring cells, the intracellular domain of Notch ligands is ubiquitylated for their processing, which is required for the maintenance and activation of Notch signaling in neighboring cells (Dutta et al., 2021). Thus, D1ICD may regulate Notch signaling not only in a cell-autonomous manner but also in a non-cell-autonomous manner.

Collectively, we propose a model in which D1ICD plays a crucial role in DRG development via two mechanisms: the modulation of the lateral inhibition mechanism by inhibiting Notch signaling; and repression of the MAP kinase pathway by inhibitingErk1/2 phosphorylation (Fig. S6B).

## MATERIALS AND METHODS

### Mice

The wild-type mice used in this study were MCH strain (CLEA Japan). Wnt1Cre and UBC CreERT2 were provided by S. Iseki (Tokyo Medical and Dental University, Tokyo, Japan) and P. Chambon (IGBMC, Illkirch, France), respectively. Transgenic mice that constitutively express loxP-CAT-loxP-3xHA_D1ICD_Flag under the control of the CAG promoter and a mouse that harbors non-cleavable Dll1 (NC-Dll1) via a specific 48 bp deletion of essential genome sequence for cleavage were generated in our laboratory by nucleotide injection into fertilized eggs. Homologous recombination was stimulated by CRISPR. Cas9 mRNA and gRNA were generated by *in vitro* transcription (Thermo Fisher Scientific) using pX330 plasmid (Addgene plasmid #50718). The gRNA sequences were CCCAGGGGAAGGGCCCGCCC and UCACUGAGGUCCACCACCAU. The Oligo DNA sequence for homologous recombination is GCTATGGCGGCCCCAACTGCCAGTTTCTGCTCCCTGAGCCACCACCAGGGCCCTTCCCCTGGGTGGCCGTGTGTGCCGGGGTGGTGCTTGTCCTCCTGCT. The NC-Dll1 allele was detected by PCR. The animals had access to a standard chow diet and water *ad libitum*, and were housed in a pathogen-free barrier facility with a 12 h light:12 h dark cycle.

### Tamoxifen injection

Pregnant mice were injected intraperitoneally with 0.5 ml tamoxifen (Sigma-Aldrich, Japan) dissolved in corn oil (20 mg/ml) at E10.5. Embryos were sampled at E12.5.

### NC-Dll1 functional analyses

Each Dll1 in pcDNA3.1 and NC-Dll1 in pcDNA3.1 was transfected into NIH3T3 cells expressing Notch1 and Lfng using Lipofectamine LTX (Thermo Fisher Scientific). 24 h after transfection, whole cells extracts were collected by Sample Buffer Solution with 2-ME (Nacalai Tesque) for western blotting. Reporter assays were carried out by the co-transfection of reporter plasmids TP1-luciferase (pGa981-6, including six copies of RBPJk-binding sites) and pRL-TK (Promega) with Dll1 or NC-Dll1 into Notch1 and Lfng-expressing NIH3T3 cells (Okubo et al., 2012). Cell lysates were then used for the luciferase assay using the Dual luciferase reporter assay system (Promega).

### Hey1 promoter assay

The Hey1 promoter construct was provided by Hiroki Kokubo (Hiroshima University, Japan). Luciferase is expressed under the control of Hey1 promoter activity. The constructs and pRL-TK (Promega) were transfected with either or both D1ICD in pc DNA3.1 and NICD in pcDNA3.1 into HEK293T cells by Lipofectamine LTX (Thermo Fisher Scientific). Cell lysates were then used for the luciferase assay using Dual luciferase reporter assay system (Promega).

### RNAi for knockdown Numb expression in NIH3T3 cells

Numb and Numbl expression was knocked down using Stealth RNAi siRNA for human Numb (HSS112687; Invitrogen) and Numbl (HSS113716), respectively. Transfection of NIH3T3 cells with the siRNA was started by the addition of Lipofectamine RNAiMAX complex (Invitrogen) containing siRNA to the culture medium. As a negative control for the siRNA treatment, Medium GC Stealth RNAi Negative Control (Invitrogen, Japan) was used.

### Immunostaining

Embryos were fixed with 4% PFA/PBS overnight at 4°C, submerged in sucrose/PBS at 4°C. The trunk of the embryo was dissected, then embedded in OCT compound (Sakura Finetek) and frozen. Following antigen retrieval, frozen sections (14 μm) were incubated with primary antibodies against cleaved Notch1 (4147S, Cell Signaling Technology; 1:1000), the Dll1C-terminus (K. Nakayama, Hokuriku University, Kanazawa, Japan; 1:1000), Dll1 N-terminus (5026, R&D Systems; 1:400) and pERK1/2 (4370, Cell Signaling Technology; 1:1000). Sections were incubated with horseradish peroxidase-conjugated donkey anti-rabbit IgG antibody (NA934, General Electric Company; 1:500) and enhanced using a Tyramide signal amplification system (Perkin-Elmer). GFP, p75, Sox10, Tuj1, BFABP and ki67 were detected using chick anti-GFP (ab13970, Abcam; 1:1000), rabbit anti-p75 (G3231, Promega; 1:500), goat anti-Sox10 (sc-17342, Santa Cruz Biotechnology; 1:100), mouse anti-Tuj1 (T8660, Sigma; 1:500), rabbit anti-BFABP (AB9558, Chemicon; 1:1000) and mouse ki67 (550609, BD Pharmingen; 1:500) primary antibodies. Bound primary antibodies were visualized using secondary antibodies: Alexa Fluor 488 goat anti-chicken IgG, Alexa Fluor 488 donkey anti-goat IgG, Alexa Fluor 488 donkey anti-mouse IgG, Alexa Fluor 488 donkey anti-rabbit IgG, Alexa Fluor 594 donkey anti-goat IgG, Alexa Fluor 594 donkey anti-mouse IgG, Alexa Fluor 594 donkey anti-rabbit IgG (A-11039, A-11055, A-21202, A-21206, A-11058, A-21203, A-21207, Thermo Fisher Scientific; 1:400). Images were acquired using the Olympus Bx51 microscope, and captured using a CCD camera and cellSens standard software (Olympus). Image settings such as brightness and contrast were changed using Photoshop CS5 extended.

### Cell counting

Because DRG development shows differences along the anteroposterior axis, counting data for comparison were collected from the sections at the thoracic level, which was determined by the appearance of the heart (Hu et al., 2011). Each score was calculated by counting each marker-positive cell of the whole DRG in one section from one embryo. The number of counted cells is shown in Table S4. The details of the quantification are described in the supplementary Materials and Methods.

### Neural crest stem cells culture and differentiation assay

Trunk DRGs at E12.5 were dissected out and digested in 0.1% collagenase (Sigma), 0.1% dispase (Roche) and 0.05% DNase I (Roche) in HBSS supplement with 10% FBS and 1% penicillin and streptomycin for 30 min at room temperature. Isolated NCSCs were cultured in DMEM/F12 medium supplemented with N2, B27, primocin, 10 ng/ml EGF, 20 ng/ml FGF, 50 ng/ml IFG and 50 ng/ml heparan sulfate on the dish coated with ornithine and fibronectin. NCSCs were plated at 1000 cells/well on an ornithine- and fibronectin-coated eight-well chamber slide, and differentiated for 3 days with DMEM/F12 supplemented with N2, B27 and primocin.

### Isolation of GFP-expressing NCSCs

NCSCs were passaged using accutase (Thermo Fisher Scientific) and filtering with strainers (40 μm). GFP-expressing NCSCs were isolated by FACS Aria (BD Biosciences). FACS data were analyzed with BD FACSDIVA software.

### Nuclear and cytoplasmic protein extraction

NCSCs were transfected with 3×HA_D1ICD_Flag in pcDNA3.1 using ViaFect transfection reagent (Promega). After 24 h, NCSCs were treated with 1 μM of the protease inhibitor epoxomicine. Nuclear and cytoplasmic proteins were extracted using NE-PER Nuclear and Cytoplasmic Extraction Reagents (Thermo Fisher Scientific).

### Immunoprecipitation

HEK293T cells were transfected with 3×Flag_D1ICD in pcDNA3.1. Transfected cells or NCSCs (±3×HA_D1ICD_Flag) were treated with IP lysis buffer (Thermo Fisher Scientific) with proteasome inhibitor cocktail (Nacalai Tesque) and immunoprecipitated with anti-Flag M2 resin or anti-HA resin (Sigma), respectively. The proteins were eluted with 3×Flag peptide (Sigma) or SDS-PAGE sample buffer (Nacalai Tesque).

### Western blotting

Immunoprecipitated proteins and NCSC lysate were separated by SDS PAGE. For immunoblotting, antibodies used were as follows: anti-HA, anti-Numb, anti-Erk1/2 and anti-pErk1/2 (2756, 4695 and 4370, Cell Signaling Technology; 1:100, 1:1000, 1:1000, 1:1000, respectively), anti-β-actin (A5316, Sigma; 1:1000), anti-Numbl, anti-Lamin B1 and anti-GAPDH (10111, 12987 and 10494, Proteintech; 1:1000, 1:2000, 1:5000, respectively), and anti-Dll1 IC-termius anti-acetylated α tubulin, anti-Cdk2 and anti-Cdk4 (sc-9102, sc-23950, sc-163 and sc-260, Santa Cruz Biotechnology; 1:100, 1:500, 1:200, 1:200, respectively). Horseradish peroxidase (HRP)-conjugated secondary antibodies were from Jackson ImmunoResearch. Each of the protein bands were visualized. For the detection of Numb, Erk1/2 and pErk1/2 protein, the same amount of each cell lysate protein was loaded. The Numb signals were calculated as fold change from D1ICD-expressing NCSCs compared with control NCSCs normalized with β-actin signal. The fold change of pErk1/2 was calculated from the phosphorylated Erk1/2 signals per Erk1/2 signal using same cell lysates.

### Immunoprecipitation and mass spectrometric analyses

To identify D1ICD-interacting proteins, HEK293T cells were transfected either with 3×Flag-tagged mouse D1ICD or empty vector, and immunoprecipitation was performed using an anti-FLAG antibody. To omit nonspecific interactants, we (1) performed two independent experiments, (2) picked up proteins reproducibly identified from both FLAG-D1ICD IPs and (3) omitted proteins identified from one of the control IPs. Thus, the resultant list of the interactants represents specific binders. Preparation for mass spectrometric analyses was performed as previously described (Ohtake et al., 2018). Proteins were separated by SDS-PAGE and stained with Bio-Safe Coomassie (Bio-Rad). The excised gel pieces were washed sequentially in 50 mM ammonium bicarbonate (NH₄HCO₃), 30% acetonitrile (ACN) for 2 h, followed by 50 mM NH₄HCO₃, 50% ACN for 1 h and 100% ACN for 15 min. Trypsin digestion was performed with 20 ng/μl modified sequence grade trypsin (Promega) in 50 mM NH₄HCO₃ and 5% ACN (pH 8.0) for 15 h at 37°C. Digested peptides were extracted in 0.1% trifluoroacetic acid (TFA) and 70% ACN four times, and concentrated by vacuum centrifugation.

Liquid chromatography mass spectrometric (LC-MS) analyses were performed essentially as previously described (Ohtake et al., 2018). A Nanoflow UHPLC, Easy nLC 1000 (Thermo Fisher Scientific), was connected online to a quadrupole-equipped Orbitrap MS instrument, Q Exactive (Thermo Fisher Scientific), with a nanoelectrospray ion source (Thermo Fisher Scientific). The Q Exactive was operated using Xcalibur software (Thermo Fisher Scientific) with data-dependent acquisition of MS2 spectra. The top 10 most intense ions with charge state of +2 to +4 were subjected to higher energy collisional dissociation (HCD) fragmentation with a normalized collision energy of 28.

The data were analyzed using Mascot in Proteome Discoverer 1.3 (Thermo Fisher Scientific, Japan). Maximum missed cleavage sites were set to 2, and the precursor and fragment mass tolerances were 10 ppm and 20 mmu, respectively. Oxidation (Met), pyroglutamate conversion (Gln) and phosphorylation (Ser, Thr and Tyr) were searched as variable modifications. Peptide identification was filtered at FDR<0.01. Gene Ontology (GO) analyses were performed using DAVID database (https://david.ncifcrf.gov/).

### Quantitative reverse transcription PCR

Total RNA was extracted from the NCSCs and pooled DRGs isolated from the thoracic and lumbar levels using RNeasy MICRO Kit (Qiagen). RNA samples were subsequently used for cDNA synthesis using rimeScript RT Reagent Kits (Takara). For quantitative PCR reactions on cDNAs, PowerUp SYBR Green Master Mix (Thermo Fisher Scientific, Japan) was used together with gene-specific primers (see Table S5).

## Supplementary Material

Supplementary information

Reviewer comments
