# Supplementary material for: Cleaved Delta like 1 intracellular domain regulates neural development via Notch signal-dependent and -independent pathways
Source: Development. 2021 Oct 4;148(19):dev193664. doi: 10.1242/dev.193664 (PMC8513606; doi:10.1242/dev.193664)
Supplement: Supplementary information [file develop-148-193664-s1.pdf]

A Dll1 protein sequence

MGRRSALALAVVSALLCQVWSSGVFELKLQEFVNKKGLLGNRNCCRGGSGPPCACRTFFRVCLKHYQASVSPEPPCTYGSAVTPV
signal peptide
LGVDSFSLPDGAGIDPAFSNPIRFPFGFTWPGTFSLIIEALHTDSPDDLATENPERLISRLTTQRHLTVGEEWSQDLHSSGRTDLRYS

YRFVCDEHYYGEGCSVFCRPRDDAFGHFTCGDRGEKMCDPGWKGQYCTDPICLPGCDDQHGYCDKPGECKCRVGWQGRYCDE
DSL EGF1
CIRYPGCLHGTCQQPWQCNCQEGWGGLFCNQDLNYCTHHKPCRNGATCTNTGQGSYTCSCRPGYTGANCELEVDECAPSPCK
EGF2 EGF3
NGASCTDLEDSFSCTCPPGFYGKVCELSAMTCADGPCFNGGRCSDNPDGGYTCHCPLGFSGFNCEKKMDLCGSSPCSNGAKCV
EGF4 EGF5 EGF6
DLGNSYLCRCQAGFSGRYCEDNVDDCASSPCANGGTCRDSVNDFSCTCPPGYTGKNCSAPVSRCEHAPCHNGATCHQRGQRY
EGF7 EGF8
MCECAQGYGGPNCQFLLPEPPPGPMVVDLSERHMESQGGPFPWVAVCAGVVLVLLLLLGCAAVVVCVRLKLQKHQPPPEPCGGE
NC-Dll1 deletion site Trans-membrane site D1ICD site
TETMNNLANCQREKDVSVSIIGATQIKNTNKKADFHGDHGAKKSSFKVRYPTVDYNLVRDLKGDEATVRDTHSKRDTKCQSQSSAG

EEKIAPTLRGGEIPDRKRPESVYSTSKDTKYQSVYVLSAEKDECVIATEV

B

Induced D1ICD — CAG promoter ◀ loxP — Chloramphenicol acetyltransferase(CAT) ◀ loxP — 3xHA_D1ICD_Flag

GFP reporter — CAG promoter ◀ loxP — Chloramphenicol acetyltransferase(CAT) ◀ loxP — EGFP

Cre recombinase — Wnt1 Cre (expressed in migrating NCCs)
or
UBC CreERT2 (inducible Cre mediated recombination by tamoxifen injection)

C

Dll1 exon9 genome sequence
GTGCCAAGTGTGTGGACCTCGGCAACTCTTACCTGTGCCGGTGCCAGGCTGGCTTCTCCGGGAGGTACTGCGAGGACAATGTGGATGACTGTGCCTCCTC
CCCGTGTGCAAATGGGGGCACCTGCCGGGACAGTGTGAACGACTTCTCCTGTACCTGCCCACCTGGCTACACGGGCAAGAACTGCAGCGCCCCTGTCAGC
AGGTGTGAGCATGCACCCTGCCATAATGGGGCCACCTGCCACCAGAGGGGCCAGCGCTACATGTGTGAGTGCGCCCAGGGCTATGGCGGCCCCAACTGC
CAGTTTCTGCTCCCTGAGCCACCACCAGGGCCCATGGTGGTGGACCTCAGTGAGAGGCATATGGAGAGCCAGGGCGGGCCCTTCCCCTGGGTGGCCGTGT
GTGCCGGGGTGGTGCTTGTCCTCCTGCTGCTGCTGGGCTGTGCTGCTGTGGTGGTCTGCGTCCGGCTGAAGCTACAGAAACACCAGCCTCCACCTGAACC
CTGTGGGGGAGAGACAGAAACCATGAACAACCTAGCCAATTGCCAGCGCGAGAAGGACGTTTCTGTTAGCATCATTGGGGCTACCCAGATCAAGAACACCA
ACAAGAAGGCGGACTTTCACGGGGACCATGGAGCCGAGAAGAGCAGCTTTAAGGTCCGATACCCCACTGTGGACTATAACCTCGTTCGAGACCTCAAGGGA
GATGAAGCCACGGTCAGGGATACACACAGCAAACGTGACACCAAGTGCCAGTCACAGAGCTCTGCAGGAGAAGAGAAGATCGCCCCAACACTTAGGGG

Fig. S1. Experimental scheme for genetically D1ICD modulating mice.
(A) Dll1 protein sequence in mouse. Orange line indicates the D1ICD sequence using CAG-floxed Chloramphenicol acetyltransferase (CAT) 3xHA_D1ICD_Flag transgenic mouse line. Blue line represents the deletion sequence for the generation of NC-Dll1 mouse to inhibit the production of D1ICD. Green line is trans-membrane sequence. (B) List of transgenic mouse lines using for D1ICD induction. (C) Genome sequence of Dll1 exon9 containing 48 bp deletion site for generation of NC-Dll1 mouse. Blue represents the deletion sequence corresponding to light blue region at (A). Black underlines indicate gRNA sequence for CRISPR-Cas9.

Development • Supplementary information

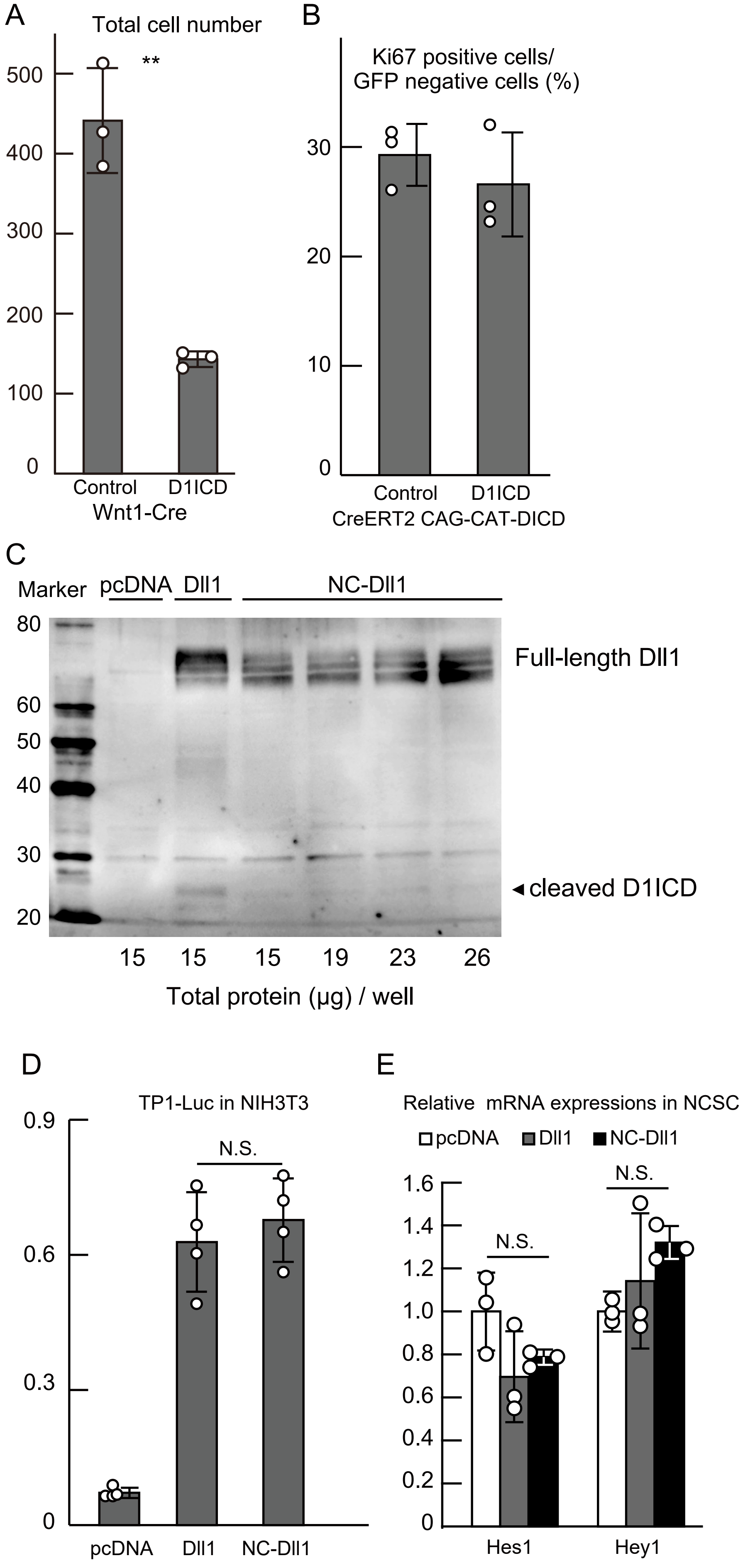

Development • Supplementary information

Fig. S2. Experimental confirmations using gene modified mice.

(A) Total cell number in DRG sections derived from CAG-floxed CAT-GFP/Wnt1-cre (control) and CAG-floxed CAT-GFP/CAG-floxed-D1ICD/Wnt1-cre (D1ICD). (B) The rate of Ki67 positive proliferating cells in GFP negative cells of DRG sections derived from CAG-floxed CAT-GFP/Cre-ERT2 (control) and CAG-floxed CAT-GFP/CAG-floxed-D1ICD/ Cre-ERT2 (D1ICD) embryos at E12.5. The induction was started at E10.5. n=3 (different animals). (C) Dll1 C-terminal protein expression by transfection of an expression vector containing wild-type- or NC- Dll1 into NIH3T3 cells expressing Notch1 and Lfng. Bottom numbers indicate total protein amounts each lane. (D) Notch signal sending abilities of transfected wild-type- or NC- Dll1 into NIH3T3 cells co-cultured with NIH3T3 cells expressing Notch1, Lfng and transfected TP1-Luc. The relative induction of luciferase activity in each sample was calculated and described as fold activation against the internal control. n=4 (independent transfection experiments). (E) mRNA expression of *Hes1* and *Hey1* in NCSCs transfected wild-type- or NC- Dll1. n=3 (independent transfection experiments). Statistical analyses were performed using the two-tailed Student’s t-test (A, B) and the one-way ANOVA with Tukey’s post hoc tests for multiple comparisons (D, E). **$p<0.01$.

Development • Supplementary information

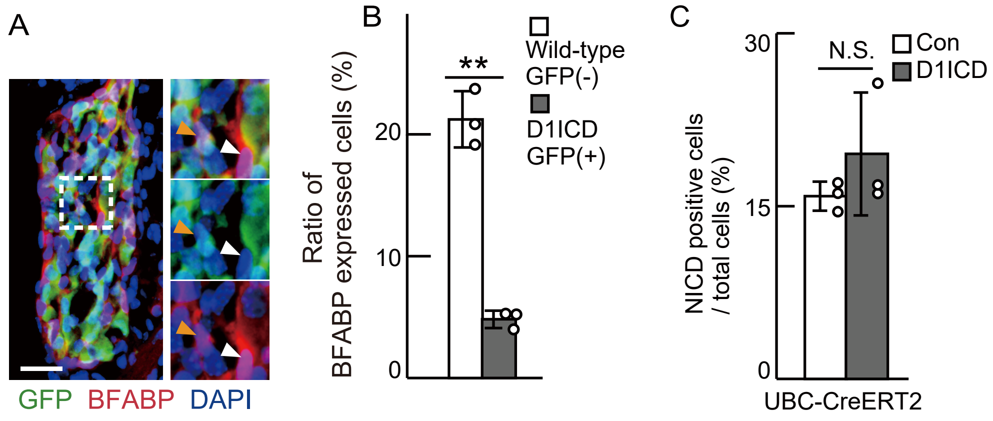

Fig. S3. Experimental confirmations for analyzing the lateral inhibition mechanism. (A, B) Immunostaining (A) and the quantification (B) of the ratio of BFABP (red) expressing cells in wild-type cells (white bar: GFP-negative) and D1ICD induced cells (gray bar: GFP-positive (green)) corresponding to (A). Each right image represents the magnification of the square area enclosed by the white dotted lines. White and orange arrows indicate BFABP-positive cells in wild-type cells and D1ICD induced cells, respectively. (C) The rate of Notch signal active cells in total cells of DRG sections derived from CAG-floxed CAT-GFP/Cre-ERT2 (control) and CAG-floxed CAT-GFP/CAG-floxed-D1ICD/ Cre-ERT2 embryos at E12.5. The induction was started at E10.5 by Tamoxifen injection. n=3 (different animals).Blue signals indicate nuclei. n=3 (different animals). Scale bars, 25 µm. Bar graphs are mean ± SD. Statistical analyses were performed using the two-tailed Student's t-test. $^{**}p<0.01$.

Development • Supplementary information

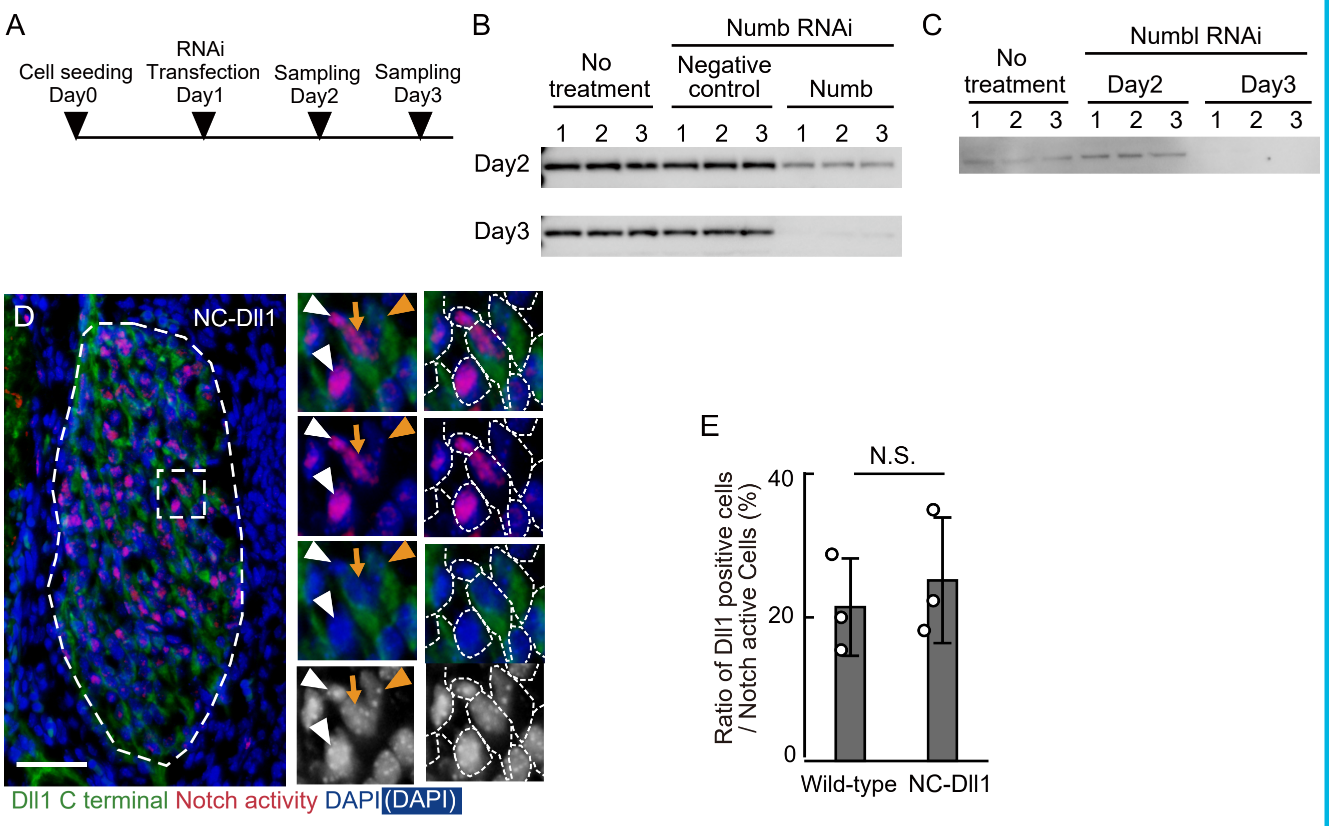

Fig. S4. Experimental confirmations for Numb/ Numbl knockdown.
(A) Experimental procedure Numb and Numbl knockdown. (B) Total Numb protein in NIH3T3 cells transfected control RNAi or Numb RNAi. (C) Total Numbl protein in NIH3T3 cells transfected Numbl RNAi. (D) Immunostaining showing Dll1 C-terminal epitope (green) and Notch activity (Red) in DRG sections derived from NC-Dll1 embryos at E12.5. Nuclei are indicated blue or white. Each right image represents the magnification of the square area enclosed by the thick white dotted lines. Thin white dotted lines represent each cell boundary. The orange and white arrowheads indicate Dll1 expressing cells and Notch active cells, respectively. The orange arrows represent the cells co-staining Dll1 and Notch activity. n=3 (different animals). (E) The ratio of NICD and Dll1 C-terminal signal co-staining cells in Notch active cells of wild-type and NC-Dll1 DRG at E12.5. n=3 (different animals, littermates). Bar graphs are mean ± SD. Statistical analyses were performed using the two-tailed Student's t-test. $^{*}p<0.05$.

Development • Supplementary information

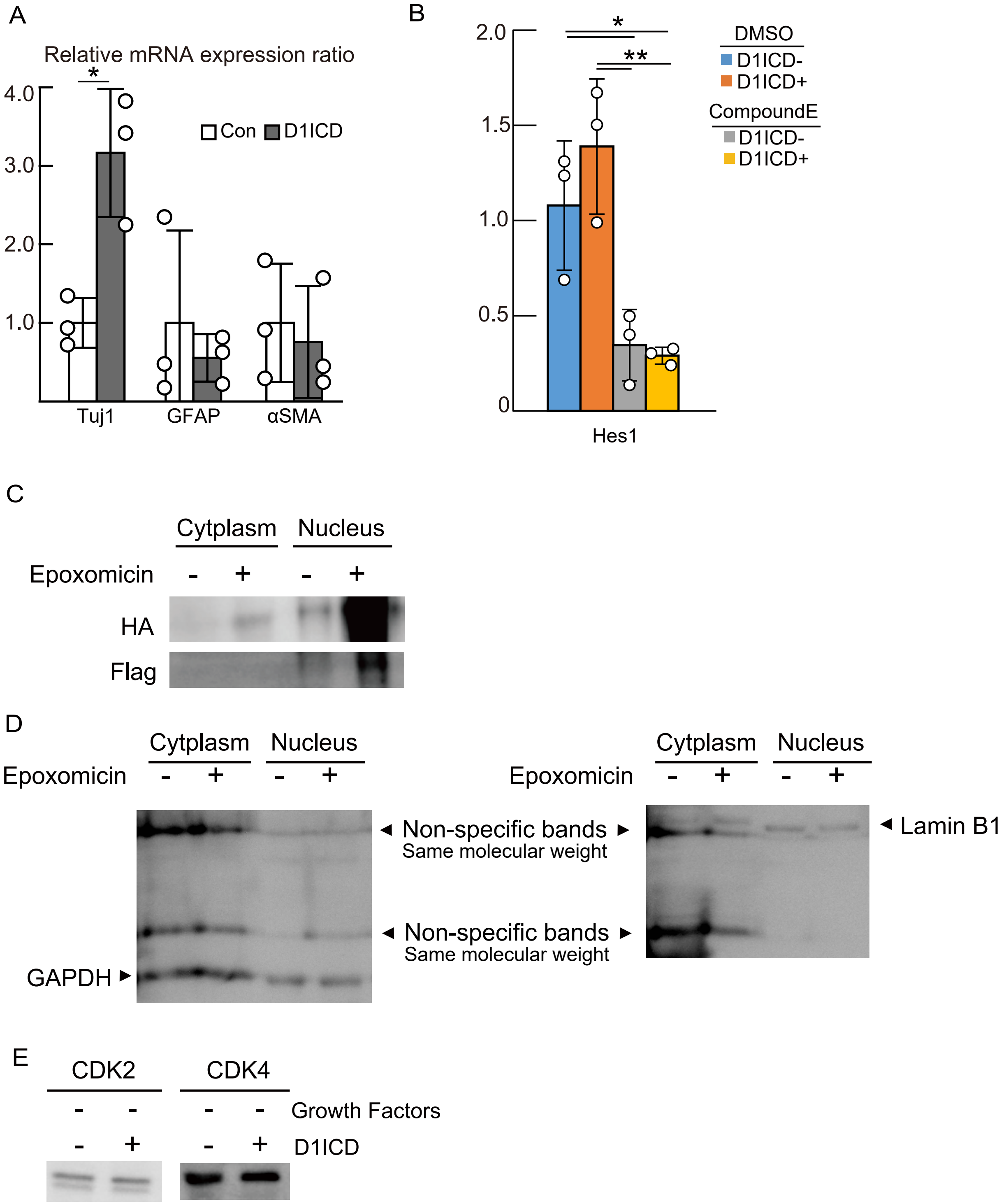
A
Relative mRNA expression ratio
*
4.0
3.0
2.0
1.0
Con
D1ICD
Tuj1
GFAP
αSMA
B
2.0
1.5
1.0
0.5
0
*
**
DMSO
D1ICD-
D1ICD+
CompoundE
D1ICD-
D1ICD+
Hes1
C
Cytplasm
Nucleus
Epoxomicin - + - +
HA
Flag
D
Cytplasm
Nucleus
Epoxomicin - + - +
Non-specific bands
Same molecular weight
Non-specific bands
Same molecular weight
GAPDH
Cytplasm
Nucleus
Epoxomicin - + - +
Lamin B1
E
CDK2
CDK4
- - - - Growth Factors
- + - + D1ICD

Fig. S5. Experimental confirmations for analysis using NCSCs.

(A) Relative differentiation marker mRNA expressions in NCSC differentiation assay using NCSC isolated from D1ICD overproduction DRG mice. (B) *Hes1* mRNA expression in NCSC differentiation assay corresponding to Fig. 8A. (C) Subcellular localization of D1ICD protein in D1ICD expressing NCSC treated with or without proteasome inhibitor Epoxomicin. (D) The Lamin B1 and GAPDH protein expression in each faction. (E) CDK2/4 protein expression in ± D1ICD induced NCSC withdrawing growth factors. Bar graphs are mean ± SD. Statistical analyses were performed using the two-tailed Student's t-test (A) and the one-way ANOVA with Tukey's post hoc tests for multiple comparisons (B). $*p<0.05$, $*p<0.01$.

Development • Supplementary information

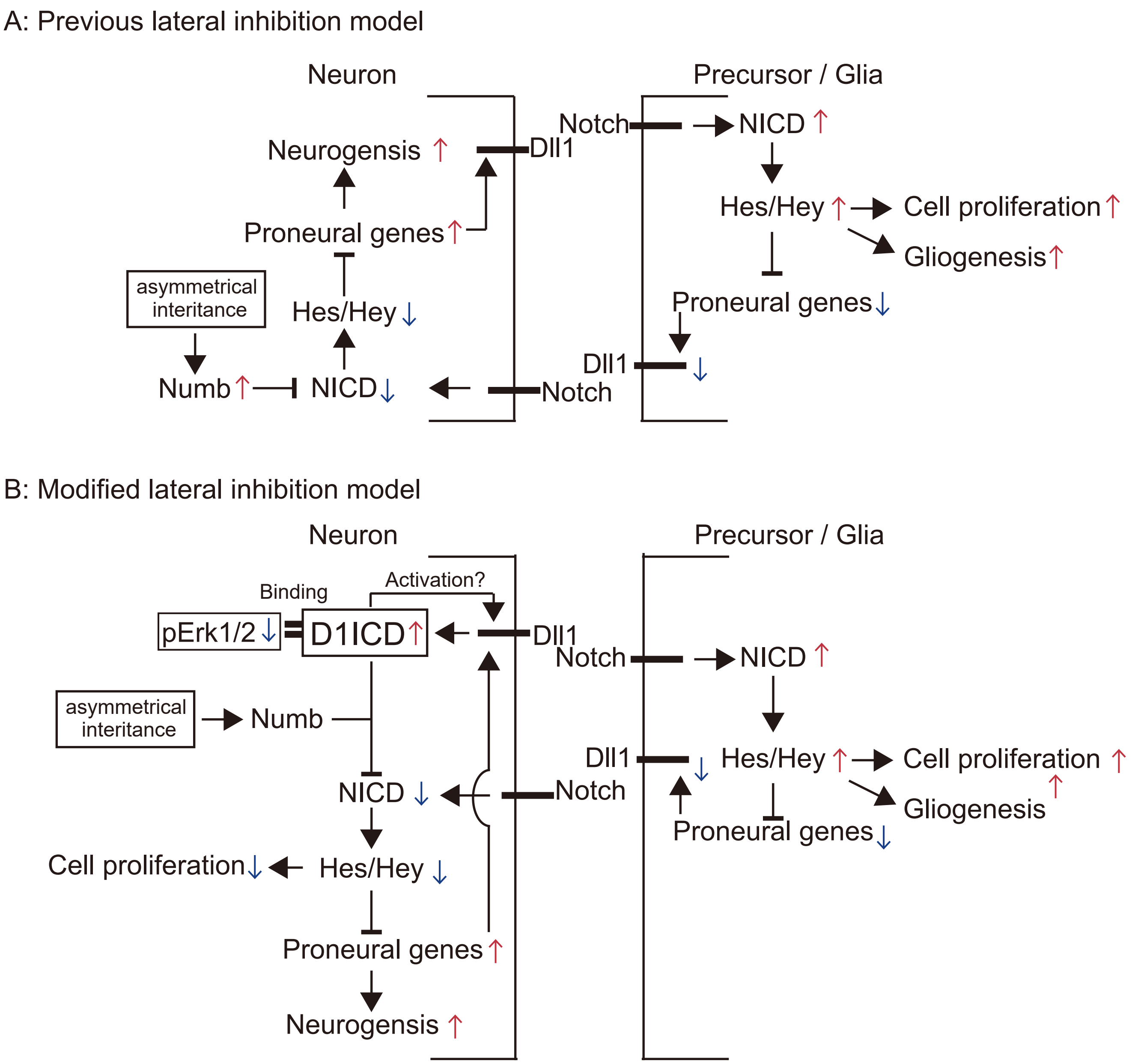

Fig. S6. Lateral inhibition models for DRG development.
(A) Previous lateral inhibition model regulated by Notch signaling. (B) Our proposal lateral inhibition model including D1ICD function which is inhibits Notch signaling and phosphorylation of Erk1/2.

Development • Supplementary information

Table S1. The 3xFlag_D1ICD binding proteins by IP-MS/MS using the anti-Flag antibody in HEK293T cells.

Click here to download Table S1

Table S2. The detail information of IP-MS/MS experiments.

Click here to download Table S2

Table S3. The 3xFlag_D1ICD binding proteins searching mouse database.

Click here to download Table S3

Table S4. The number of cell counting.

Click here to download Table S4

Table S5. Primers

	Forward (5′–3′)	Reverse (5′–3′)
Tuj1[1]	TGGACAGTGTTCGGTCTGG	CCTCCGTATAGTGCCCTTTGG
GAFAP[1]	GGGACAACTTTGCACAGGAC	GCTTCATCTGCCTCCTGTCT
a-SMA[2]	CTGACAGAGGCACCACTGAA	CATCTCCAGAGTCCAGCACA
Numb[3]	AAAGCAGTGAAGGCCGTTCT	GTTTTCTCGTCCACAACTCTGAG
Numbl[4]	GCAGGCACCATGAACAAGTTA	TCTTCACAAACGTGCATTCCC
Gapdh[2]	ACTTTGTCAAGCTCATTCC	TGCAGCGAACTTTATTGATG
Hes1[5]	CCAGCCAGTGTCAACACGA	AATGCCGGGAGCTATCTTTCT
Hey1[6]	GCGCGGACGAGAATGGAAA	TCAGGTGATCCACAGTCATCTG
Dll1[7]	CAGGACCTTCTTTCGCGTATG	AAGGGGAATCGGATGGGGTT

1. Larzabal, L., El-Nikhely, N., Redrado, M., Seeger, W., Savai, R., Calvo, A. (2013). Differential effects of drugs targeting cancer stem cell (CSC) and non-CSC populations on lung primary tumors and metastasis. *PLoS ONE* **8**, e79798.

2. Srivastava, R., Kumar, M., Peineau, S., Csaba, Z., Mani, S., Gressens, P., El Ghouzzi, V. (2013). Conditional induction of Math1 specifies embryonic stem cells to cerebellar granule neuron lineage and promotes differentiation into mature granule neurons. *Stem Cells* **31**, 652-665.

3. PrimerBank ID: 12835800a1.

4. PrimerBank ID: 6754914a1.

5. PrimerBank ID: 6680205a1.

6. PrimerBank ID: 6754188a1.

7. PrimerBank ID: 6681197a1.

Development • Supplementary information

Supplementary Materials and Methods

Signal-positive cells were counted using the following criteria: Each cell was stained with DAPI. The proteins localized in the nucleus, such as Ki67, Sox10, NICD, and pErk1/2, were counted only by co-staining with DAPI. Proteins expressing a small population, such as cleaved caspase 3 and p75, were clearly discriminated by the cell. GFP protein is recognized as a distinct single cell by staining with anti-GFP antibody (ab13970, Abcam) and DAPI. Therefore, GFP reporter-expressing cells crossing the Wnt1Cre line or UBC-CreERT2 line can also be counted using antibodies against each marker protein. Tuj1 and BFABP expression were found mainly in the cytoplasm. To clearly distinguish between cells expressing these proteins in wild-type and NC-Dll1 DRGs, only cells in which cytoplasmic staining clearly surrounded or covered the nucleus were counted as a single positive cell. Dll1 was also expressed predominantly in the cytoplasm. The cell boundaries were determined by Dll1 staining of the cytoplasm and DAPI staining of the nuclei. GFP-negative cells surrounding GFP-positive cells were discriminated using GFP staining. Because whole GFP-positive cells were distinguishable as single cells, GFP-negative cells surrounding GFP-positive cells were distinguished using DAPI staining surrounding GFP-positive cells within a distance of 1.5-fold diameter of the nucleus in the GFP-positive cells from the outline of GFP-positive cells.

Development • Supplementary information
