# Supplementary material for: Cleaved Delta like 1 intracellular domain regulates neural development via Notch signal-dependent and -independent pathways
Source: Development. 2021 Oct 4;148(19):dev193664. doi: 10.1242/dev.193664 (PMC8513606; doi:10.1242/dev.193664)
Supplement: Reviewer comments [file develop-148-193664_review_history.pdf]

Cleaved Delta like 1 intracellular domain regulates neural development via Notch signal-dependent and -independent pathways

Yusuke Okubo, Fumiaki Ohtake, Katsuhide Igarashi, Yukuto Yasuhiko, Yoko Hirabayashi, Yumiko Saga and Jun Kanno
DOI: 10.1242/dev.193664

Editor: Francois Guillemot

Review timeline
Original submission: 4 June 2020
Editorial decision: 13 July 2020
First revision received: 25 May 2021
Editorial decision: 14 June 2021
Second revision received: 2 September 2021
Accepted: 6 September 2021

Original submission

First decision letter

MS ID#: DEVELOP/2020/193664

MS TITLE: Notch-reverse signaling regulates neural development via modulating both Notch and ErbB signalings

AUTHORS: Yusuke Okubo, Fumiaki Ohtake, Katsuhide Igarashi, Yukuto Yasuhiko, Yoko Hirabayashi, Yumiko Saga, and Jun Kanno

I have now received the reports of three referees on your manuscript and I have reached a decision. The referees' comments are appended below, or you can access them online: please go to BenchPress and click on the 'Manuscripts with Decisions' queue in the Author Area.

As you will see, all the referees express great interest in your work, but they also have significant criticisms and recommend a substantial revision of your manuscript before we can consider publication. In particular, they recommend that you use additional antibodies to distinguish endogenous Dll1 from overexpressed D1ICD, that you provide details on the method to quantify immunofluorescence and demonstrate that your quantification is unbiased, that you include additional controls to some of your experiments, and that you provide further evidence that conventional Dll1-Notch1 is not affect in experiments where Dll1 cleavage is blocked. However it does not seem necessary that you analyse other regions of the nervous system of the DC-Dll1 miceas suggested by referee 1. If you are able to revise the manuscript along the lines suggested, which may involve further experiments, I will be happy to receive a revised version of the manuscript. Your revised paper will be re-reviewed by the original referees, and its acceptance will depend on your addressing satisfactorily all their major concerns. Please also note that Development will normally permit only one round of major revision.

We are aware that you may currently be unable to access the lab to undertake experimental revisions. If it would be helpful, we encourage you to contact us to discuss your revision in greater detail. Please send us a point-by-point response indicating where you are able to address concerns raised (either experimentally or by changes to the text) and where you will not be able to do so

© 2021. Published by The Company of Biologists under the terms of the Creative Commons Attribution License (https://creativecommons.org/licenses/by/4.0/).

within the normal timeframe of a revision. We will then provide further guidance. Please also note that we are happy to extend revision timeframes as necessary.

Please attend to all of the reviewers' comments and ensure that you clearly highlight all changes made in the revised manuscript. Please avoid using 'Tracked changes' in Word files as these are lost in PDF conversion. I should be grateful if you would also provide a point-by-point response detailing how you have dealt with the points raised by the reviewers in the 'Response to Reviewers' box. If you do not agree with any of their criticisms or suggestions please explain clearly why this is so.

Reviewer 1

Advance summary and potential significance to field

The authors sought to elucidate the function of the intracellular domain of Delta-like1 (D1ICD) in the developing dorsal root ganglia (DRG) by generating Dll1-overexpressing and NC-Dll1 mice. Interestingly, D1ICD overexpression inhibited cell proliferation and accelerated neurogenesis whereas inhibition of D1ICD formation (NC-Dll1) decreased neurogenesis and increased the DRG cell number. D1ICD inhibited Notch signaling partly by inducing Numb, which could act as a Notch inhibitor. Furthermore, D1ICD inhibited phosphorylation of Erk1/2 by physical interaction and thereby promoted neuronal differentiation. This is an interesting work showing the in vitro and in vivo functions of D1ICD.

Comments for the author

While the authors analyzed the in vitro and in vivo functions of D1ICD in detail, the mechanistic analyses are still preliminary and need more clarification. Specific comments are indicated below.

1. Fig. 1A showed that Dll1 and NICD expression are mutually exclusive in the developing DRG. Here, Dll1 expression seemed mostly cytoplasmic, but did the authors use the Dll1 C-terminal-specific antibody? If this is the case, did they detect the nuclear expression?

2. In lines 205-206, the authors stated that the C-terminal-specific antibody recognized both endogenous Dll1 and exogenous D1ICD, but it is not clear how they differentiated between the endogenous and exogenous Dll1 expression. Because Dll1 antibodies that can recognize the extracellular domain are commercially available (for example, R&D Systems AF3970), the authors should use such antibodies to avoid the signals from the overexpressed transgene. They also stated that when D1ICD was overexpressed, Dll1 signal was not detected in the GFP-negative cells (lines 206-207), but this could be due to the inability of the authors' antibody to detect the endogenous expression under this condition. The authors should use the one that can detect only the endogenous Dll1 here.

3. Western blot analysis showed that D1ICD is present in the nuclei of D1ICD-overexpressing cells (Fig. S3G). However, Fig. 4 indicated that D1ICD is present mostly in the cytoplasm. The authors should show better immunostaining data.

4. Fig. 4I showed the increase of NICD-expressing cells in the NC-Dll1 DRG. Here, Dll1 and NICD could be co-expressed, and the authors should examine this possibility.

5. In line 225, the authors stated that DRG neurogenesis does not occur in Numb KO mice, citing reference #21, but this reference does not show such data. It was previously demonstrated that in the absence of Numb and Numbl, neurogenesis occurs normally whereas axonal arborization is severely reduced in the DRG (Huang et al. Genes Dev. 19, 138-151, 2005). Thus, the statement and reference should be corrected.

6. In lines 234-235, the authors suggested that Numb could inhibit Notch activity, but the reference #23 indicated that Numb and Numbl cooperatively promote the maintenance of neural progenitors, suggesting that mammalian numb homologues could activate Notch signaling. In Drosophila, Numb inhibits Notch signaling by promoting Notch endocytosis, so even if mammalian Numb acts as a Notch inhibitor in some contexts, like Drosophila numb, I am not sure whether Numb can

© 2021. Published by The Company of Biologists under the terms of the Creative Commons Attribution License (https://creativecommons.org/licenses/by/4.0/).

antagonize NICD overexpression, as suggested in Fig. 5D. The authors should clarify this issue and examine the mechanism of how Numb can antagonize NICD overexpression.

7. In Fig. 5D, the authors should explain the conditions of colored bars.

8. The authors stated that homozygous NC-Dll1 mice showed no obvious abnormality in line 132. While I understand that this study focused on the analysis of DRG, the authors should briefly show their representative morphology and histology of the other nervous system, because this is the first report.

Reviewer 2

Advance summary and potential significance to field

Okubo et al, sought to identify the biological function of delta like 1 intercellular domain (D1ICD). The authors combine in vivo and in vitro approaches by generating mouse lines that overexpress the intracellular domain of the delta like receptor thus mimicking its activation, or by generating mouse lines with a Delta receptor mutant modified in such a way that inhibits cleavage of intracellular domain thus disabling activation of the pathway. By comparing those mouse lines they present a model in which activated DLL1 signaling modulates both Notch and ErbB signaling.

There is plethora of information about the impact of Notch signaling in development that shows its crucial role during the early stages of tissue specialization. However the role of the Delta 1 transmembrane receptor, known primarily as a ligand activating Notch signaling, is understudied by comparison.
The approach of the authors to combine analysis of the in vivo complementary mouse lines with results obtained from in vitro studies is pertinent and is potentially beneficial for the field. The data and methodology are appropriate for the journal.

Comments for the author

However, there are few major conceptual questions focusing on the “appropriate controls” in this manuscript. The authors do not provide valid controls for their mouse line, immunofluorescence experiments and in vivo experiments. Combined these points could lead to possible false-positive results for interpreting the impact of DLL1 on regulation of both Notch and ErbB signaling pathways. My recommendation is to better validate the assumptions and findings, which would strengthen the results and possibly further improve our understanding of DLL1 signaling.

Major concerns.
1. The authors claim that Wnt1-cre mice are specific for the neural crest cells (NCCs). They correctly show recombination in control and D1ICD knock-in mouse. However, they fail to show that this cell line is specific for the NCCs cells at this stage of the organism development. Wnt1 signaling is extremely important in the context of many tissues during early developmental stages therefore proving that that this mouse line affects only the cells of interest is crucial for the well-rounded assumption that leads towards their interpretation of their data. If this study was already conducted, authors should cite the papers but they should also demonstrate that the system works in their hands.
Immunohistochemistry, immunofluorescence and/or a combination of FACS and Western blotting would be sufficient to show that GFP+ cells are indeed overexpressing D1ICD. This data would definitively confirm that the phenotype observed by the authors comes from the changes in D1ICD levels in NCC and not from altered signaling at a systemic level.

2. The authors claim activation of inducible mouse (CRE-ERT) based on tamoxifen injection. Moreover, they state that they can induce cre activity sparsely by decreasing tamoxifen concentration. The authors fail to demonstrate the cre efficiency within the specified area, or a larger fragment of the organism. A number of methods already available to the authors can directly address this concern.

© 2021. Published by The Company of Biologists under the terms of the Creative Commons Attribution License (https://creativecommons.org/licenses/by/4.0/).

3. The authors do not mention or disclose control data (or its existence) in the biochemical experiments. The authors of this paper perform an immunoprecipitation of D1ICD and its interactors in HEK293T cells (human), because the pull down from their mutant mouse cells do not express enough of D1ICD (line 278-280;). However, in the previous fractionation experiment they determine the localization of substantial amounts of D1ICD in NCSCs (Figure S3G). The authors of the paper do not mention IgG control for the IP:D1ICD or an alternative control.
Are the interactomes in the table EV1 representing all the proteins detected by mass spec? Are these listed proteins those that were specific for the IP:D1ICD as opposed to the IP:IgG control? This section is unclear. Furthermore, the authors do not show that they detect the D1ICD in the samples. This result questions the validity of their interactome, as the authors interpret their pulldown as successful based upon finding 2 proteins that have been previously been shown to interact with D1ICD.

4. Finally, the title of the paper claims that they have revealed modulation of ErbB signaling, however the authors show only the changes in phosphorylated Erk1/2. The authors then state that pErk1/2 acts as an effector of Neuregulin-ErbB signaling. They claim (line 321-323) to SHOW a functional link between Notch-Delta and NRG1-ErbB signaling pathways. The assumption that pErk1/2 is, in this example specific to ErbB signaling is overinterpreted because the Erk1/2 molecule is an effector in a number of different signaling pathways. No data on neuregulin has been shown in this manuscript.

Minor concerns:
1. Authors of this manuscript must enclose information as to whether the mutant mice are littermates of the corresponding controls. Since there are significant size differences in the tissues, it is important to know the relationship of the compared mice.

2. In several areas the grammar needs to be corrected – sometimes entire fragments of the manuscript are extremely difficult to understand such as line 59 line 170, line 182, lines 189-197. In general many sections are excessively wordy with sentences that are too long.

3. Authors should show that the fractionation experiment was successful by proper controls for each fraction (fig S3G), e.g. GAPDH, lamin B1, etc.

4. Given the lack of controls in previous parts I would like to see controls showing that the Erk1/2 immunostaining (Fig 6D) is specific.

Reviewer 3

Advance summary and potential significance to field

This paper examines whether the intracellular domain (D1ICD) of the Notch ligand Delta1 regulates signalling and differentiation cell autonomously in addition to the more conventional function of Delta to signal through Notch to neighbouring cells. It has previously been shown that mice can develop apparently normally when engineered to suppress cleavage of Delta1. However this does not exclude the possibility that D1ICD influences development in relatively subtle ways that are not essential for viability.

In the current report the authors provide evidence that suppressing cleavage of D1ICD disrupts the normal balance of proliferation and differentiation of neural progenitors in the DRG. These loss-of-function experiments are supported by gain-of-function studies in which D1ICD is constitutively expressed within the DRG.

Overall these data look promising, and if confirmed will be important for the Notch field, but are difficult to interpret with confidence for reasons outlined in "Suggestions to authors".

Comments for the author

Overall comments

© 2021. Published by The Company of Biologists under the terms of the Creative Commons Attribution License (https://creativecommons.org/licenses/by/4.0/).

1) Does D1ICD really have an 'unconventional' autonomous intracellular role as the authors propose? An alternative possibility is that their experimental manipulations of Dll1 are interfering artificially with the conventional function of Dll1 to signal to neighbouring cells through Notch (already known to influence differentiation in the DRG). This would then indirectly result in changes to the Delta-manipulated cells due to Notch-mediated lateral inhibition.
The authors go some way towards supporting their intepretation (i.e a distinct cell-autonomous role for D1ICD) by showing that uncleavable Dll1 is able to activate Notch in NIH3T3 cells using a luciferase assay. However it seems important to provide evidence that "conventional" juxtacrine function of Dll1 is not artefactually compromised or over-stimulated when they suppress Dll1 cleavage *in DRGs*, particularly given their finding in Fig4 that D1ICD does actually seem to influence conventional Notch signalling in neighbouring cells.

2) Quantification of phenotypes The authors document phenotypes by quantifying immunofluorescence in sections of DRGs. The message of the paper relies entirely on these quantifications because there is no reported functional defect in the mice (other than anecdotal mention of hyperactivity, data not shown). However there is no information on how quantification is performed- the materials and methods states that quantification was performed by counting cells in Photoshop rather than by using computational segmentation algorithms. It is not stated how many cells were counted from how many sections of how many DRGS, nor is it stated what criteria were used for scoring cells. Were sections scored blinded or not? I'm concerned that the methods used may be subject to inadvertent user bias. Given the relatively subtle nature of some of the phenotypes I think this raises a major concern about the reliability of the quantification on which the reported phenotypes are based.
The authors could confirm their findings with an unbiased approach such as analysis of gene expression in acutely isolated cells from the DRG. They already describe experiments in which Delta-manipulated cells are sorted from the DRG and cultured in vitro for further phenotypic analysis, but it is important that they also analyse gene expression in *acutely* isolated cells in order to back up their immunofluorescence phenotyping data.

3) Mechanism It is unclear whether effects on Erk and Numb are direct consequence of interaction with Dl1 or an indirect consequence of changes to conventional Notch activity and subsequent changes to proliferation and differentiation.
I suggest that Erk activity and Numb expression should be measured in Dl1-manipulated cells isolated acutely from the DRG (rather than after a period of cell culture). Even if successful, this still leaves open the possibility of indirect effects and so this should be discussed as a caveat within the paper

Additional comments
Fig 2D/E please show TUJ1 staining alone in the DRG (i.e. not overlaid with GFP) to make it easier to compare whether or not there is really more TUJ1 in D1ICD DRGs. In the images shown the higher levels of GFP in controls tend to obscure the TUJ1 staining.
Fig 2 G-J Several markers are shown in G-J but it is difficult to see how many cells are positive for these markers because the large number of orange arrows obscure the image. Can the images be shown in a way that makes it easier for us to compare?
Fig4 looks at cell autonomous vs. non autonomous effect using low-dose tamoxifen to induce DlCD in a few cells. There is some evidence that D1ICD cells are found next to cells with activated Notch, but we are not told how many cells are scored or how it is determined objectively whether a cell is a neighbour to a green cell. I found it difficult to understand how to reliably interpret these data.
Fig5: I found it difficult to understand the rationale for the experimental design here. If the claim is that Dl1ICD activates Numb why is there no analysis of Numb expression in vivo in D1ICD-overexpressing DRGS or uncleavable-D1 DRGS? Or in acutely isolated cells from those DRGs? We are only shown analysis of cells after isolation and culture.
5A: If the authors wish to convince us that their isolated DRG cells can differentiate into three lineages I’d suggest that they do more than just show us a single example of a cell from each lineage.
5D shows an experiment in 3T3 cells that seems to be designed to test whether Numb is required for Dl1ICD to influence Notch target genes based on a luciferase assay. I found this impossible to understand- the graph is not labelled and not explained in the legend.

© 2021. Published by The Company of Biologists under the terms of the Creative Commons Attribution License (https://creativecommons.org/licenses/by/4.0/).

Fig6: The authors use an assay based on 'isolated' single cells to exclude the possibility that Dl1ICD is influencing juxtacrine signalling in this experimental setup. I found this unconvincing- neural progenitors should divide and generate neighbours for themselves in culture, and if they do not then they are probably either damaged cells or differentiated cells rather than neural progenitors. Furthermore, it is not stated how many cells were scored and how many cells were excluded from this assay.
Fig6: The authors should discuss the possibility that the observed differences in pErk may be an indirect consequence of changes in proliferation/differentiation rather than an upstream cause of these changes.

First revision

Author response to reviewers' comments

MS TITLE:
After revision: Cleaved Delta like 1 intracellular domain regulates neural development via Notch signal dependent and independent pathways
Before revision: Notch-reverse signaling regulates neural development via modulating both Notch and ErbB signalings

AUTHORS: Yusuke Okubo, Fumiaki Ohtake, Katsuhide Igarashi, Yukuto Yasuhiko, Yoko Hirabayashi, Yumiko Saga, and Jun Kanno

In summary, we have performed new experiments, as requested by multiple reviewers. We succeeded in distinguishing endogenous Delta-like 1 (Dll1) from overexpressed Dll1 intracellular domain (D1ICD) using an additional antibody. We have also provided detailed methods to explain quantification. We also analyzed gene expression in acutely isolated DRGs from wild-type and Non-cleavable-Dll1 (NC-Dll1) embryos to support the quantification analysis by cell counting. To confirm that NC-Dll1 does not influence conventional Dll1-Notch1 signaling, we examined Notch target gene mRNA expression in NCSCs. The results suggest that the Notch signal sending ability was not different between wild-type-Dll1 and NC-Dll1. Previously, we showed that D1ICD promotes neuronal differentiation even in low-density culture conditions where Notch-Delta signal interaction is inhibited. To obtain more direct evidence, we conducted a differentiation assay on the NCSC in the presence of a γ-secretase inhibitor and confirmed that overexpressed D1ICD promoted neuronal differentiation in the absence of endogenous Notch-Delta signaling. Moreover, we confirmed the nuclear fractionation of NCSC by western blotting using an anti-Lamin B1 antibody. Finally, we have changed the title and parts of the manuscript as suggested by the reviewers.

We are grateful for the reviewer's very helpful suggestions which led us to perform these additional experiments and also make the appropriate change to the manuscript, which we feel has significantly improved the paper. The text below provides our detailed response, highlighted in bold, following each individual suggestion/comment of the corresponding reviews of each reviewer. We have also highlighted these revisions in the revised manuscript.

Reviewer #1:
While the authors analyzed the in vitro and in vivo functions of D1ICD in detail, the mechanistic analyses are still preliminary and need more clarification. Specific comments are indicated below.

1. Fig. 1A showed that Dll1 and NICD expression are mutually exclusive in the developing DRG. Here, Dll1 expression seemed mostly cytoplasmic, but did the authors use the Dll1 C-terminal-specific antibody? If this is the case, did they detect the nuclear expression?

Yes, we have used a Dll1 C-terminal-specific antibody. However, despite of using an antibody to recognize Dll1 C-terminal, the Dll1 signal was hardly detected in nuclei. It has been reported that nuclear D1ICD is degraded by proteasomes rapidly, (E. Six et al., PNAS. 100, 7638-7643,

© 2021. Published by The Company of Biologists under the terms of the Creative Commons Attribution License (https://creativecommons.org/licenses/by/4.0/).

2003; E. Dyczynska et al., JBC. 282, 436-444, 2007); hence, we tested for this by treatment with the protease inhibitor Epoxomicin. We observed that the D1ICD protein levels increased in the nuclei (Fig.S5 B), supporting the idea that the D1ICD is quickly degraded once it enters into the nuclei of the DRG, thus supporting our initial observation of detecting lesser nuclear expression of D1ICD.

2. In lines 205-206, the authors stated that the C-terminal-specific antibody recognized both endogenous Dll1 and exogenous D1ICD, but it is not clear how they differentiated between the endogenous and exogenous Dll1 expression. Because Dll1 antibodies that can recognize the extracellular domain are commercially available (for example, R&D Systems AF3970), the authors should use such antibodies to avoid the signals from the overexpressed transgene. They also stated that when D1ICD was overexpressed, Dll1 signal was not detected in the GFP-negative cells (lines 206-207), but this could be due to the inability of the authors' antibody to detect the endogenous expression under this condition. The authors should use the one that can detect only the endogenous Dll1 here.
We succeeded in detecting the endogenous Dll1 by using an antibody recommended by the reviewer (R&D Systems, AF5026). We have also confirmed that the endogenous Dll1 signal was not detected in GFP-negative cells (Fig.5 E, F).

3. Western blot analysis showed that D1ICD is present in the nuclei of D1ICD-overexpressing cells (Fig. S3G). However, Fig. 4 indicated that D1ICD is present mostly in the cytoplasm. The authors should show better immunostaining data.
Dll1 signal in Fig.4 before revision detected not only overexpressed D1ICD but also endogenous full-length Dll1. As explained in the reply to the first suggestion, the cleaved D1ICD is hardly detected in nuclei by immunostaining. Instead of the immunostaining, we have shown nuclear localization of D1ICD by western blotting (Fig. S5 B).

4. Fig. 4I showed the increase of NICD-expressing cells in the NC-Dll1 DRG. Here, Dll1 and NICD could be co-expressed, and the authors should examine this possibility.
We had shown that the ratio of Notch-positive cells to total cells is increased in NC-Dll1 compared to wild-type DRGs. Here, we found that the ratio of cells co-stained with NICD and Dll1 C-terminal epitope in NICD-positive cells did not change significantly between wild-type and NC-Dll1 DRGs (Fig. S4 D, E). The ratio of the co-stained cells to total cells however, was significantly increased in NC-Dll1 compared to wild-type DRGs (Fig. S4 F). This result suggests that the repression of Dll1 expression by Notch signalling through the lateral repression mechanism is also applicable in NC-Dll1 DRGs. These results indicate that the increased Notch signal-positive cell ratio in NC-Dll1 DRGs may be due to the disruption of cell-autonomous Notch signal suppression by D1ICD since the Notch signal sending ability of Wild-type and NC-Dll1 is unchanged in both NIH3T3 and NCSCs (Fig. S2D, E, S4 G).

5. In line 225, the authors stated that DRG neurogenesis does not occur in Numb KO mice, citing reference #21, but this reference does not show such data. It was previously demonstrated that in the absence of Numb and Numbl, neurogenesis occurs normally whereas axonal arborization is severely reduced in the DRG (Huang et al. Genes Dev. 19, 138-151, 2005). Thus, the statement and reference should be corrected.
We apologize for our mistake. We intended to refer to a paper that reported that NeuroD is not expressed in the DRGs of conventional Numb KO embryos at E10.5 and that neurogenesis does not occur (M. Zirlinger et al., Proc Natl Acad Sci U S A 99, 8084-8089 (2002)). We have revised our statement and also have cited the appropriate paper (Huang et al. Genes Dev. 19, 138-151, 2005).
Before revision:
It has been reported that during cell division, the asymmetric distribution of Numb protein, which works as a Notch signaling inhibitor, regulates cell fate decision in the developing chick DRG (1), and that DRG neurogenesis does not occur in the Numb knockout mice (M. Zirlinger et al., Proc Natl Acad Sci U S A 99, 8084-8089 (2002)).
After revision:
It has been reported that during cell division, the asymmetric distribution of Numb protein, which works as a Notch signaling inhibitor, regulates cell fate decision in the developing chick DRG (1), and that Numb inhibits NICD nuclear localization in isolated mouse DRG cells (Huang et al. Genes Dev. 19, 138-151, 2005).

© 2021. Published by The Company of Biologists under the terms of the Creative Commons Attribution License (https://creativecommons.org/licenses/by/4.0/).

6. In lines 234-235, the authors suggested that Numb could inhibit Notch activity, but the reference #23 indicated that Numb and Numbl cooperatively promote the maintenance of neural progenitors, suggesting that mammalian numb homologues could activate Notch signaling. In Drosophila, Numb inhibits Notch signaling by promoting Notch endocytosis, so even if mammalian Numb acts as a Notch inhibitor in some contexts, like Drosophila numb, I am not sure whether Numb can antagonize NICD overexpression, as suggested in Fig. 5D. The authors should clarify this issue and examine the mechanism of how Numb can antagonize NICD overexpression.
We apologize for the incomplete figure 5D. We found that Hey1 promoter activity induced by NICD was increased in both Numb and Numbl knockdown (Fig. 6B).
In addition, there are many reports that Numb inhibits Notch signaling. In the reference #23 which reviewer mentioned, loss of Numb and Numbl reduces endocytosis and leads to a modest increase in nuclear Notch1 in isolated DRG cells (Huang et al. Genes Dev. 19, 138-151, 2005). Mammalian Numb promotes the ubiquitination of membrane-bound Notch1 through an E3 ligase-dependent mechanism, which leads to degradation of Notch1 ICD and loss of Notch-dependent transcriptional activation of Hes1 (McGill and McGlade. JBC. Vol. 278, No. 25, Issue of June 20, pp. 23196-23203, 2003). Numb and Numblike down-regulate NICD protein levels (Chapman et al., JCB. Vol. 175, No. 4, November 20, 2006 535-540. These results suggest that Numb also inhibits Notch signaling in mouse DRG by decreasing nuclear NICD.
We have also added above reference in the manuscript:
It has been reported that during cell division, the asymmetric distribution of Numb protein, which works as a Notch signaling inhibitor (Chapman et al., 2006; McGill and McGlade, 2003), regulates cell fate decision in the developing chick DRG (Wakamatsu et al., 2000), and that Numb inhibits NICD nuclear localization in isolated mouse DRG cells (Huang et al., 2005).

7. In Fig. 5D, the authors should explain the conditions of colored bars.
We apologize for the incomplete figure. As explained in our reply to the above comment number 6, we have explained the condition of colored bars.

8. The authors stated that homozygous NC-Dll1 mice showed no obvious abnormality in line 132. While I understand that this study focused on the analysis of DRG, the authors should briefly show their representative morphology and histology of the other nervous system, because this is the first report.
We sincerely appreciate the kind suggestion. As the reviewer has noted, we are also interested in the role of D1ICD in other tissue including central nervous system. We are currently analyzing the role of D1ICD in the central nervous system and will present the results in the near future.

Reviewer #2:
However, there are few major conceptual questions focusing on the “appropriate controls” in this manuscript. The authors do not provide valid controls for their mouse line, immunofluorescence experiments and in vivo experiments. Combined, these points could lead to possible false-positive results for interpreting the impact of DLL1 on regulation of both Notch and ErbB signaling pathways. My recommendation is to better validate the assumptions and findings, which would strengthen the results and possibly further improve our understanding of DLL1 signaling.

Major concerns.
1.The authors claim that Wnt1-cre mice are specific for the neural crest cells (NCCs). They correctly show recombination in control and D1ICD knock-in mouse. However, they fail to show that this cell line is specific for the NCCs cells at this stage of the organism development. Wnt1 signaling is extremely important in the context of many tissues during early developmental stages therefore proving that that this mouse line affects only the cells of interest is crucial for the well-rounded assumption that leads towards their interpretation of their data. If this study was already conducted, authors should cite the papers but they should also demonstrate that the system works in their hands. Immunohistochemistry, immunofluorescence and/or a combination of FACS and Western blotting would be sufficient to show that GFP+ cells are indeed overexpressing D1ICD. This data would definitively confirm that the phenotype observed by the authors comes from the changes in D1ICD levels in NCC and not from altered signaling at a systemic level.
The Wnt1Cre transgenic line was widely used to generate NCCs derivatives specific gene deletion and activation via Cre-loxP recombination system (i.g., T. J. Mead, Developmental

© 2021. Published by The Company of Biologists under the terms of the Creative Commons Attribution License (https://creativecommons.org/licenses/by/4.0/).

Dynamics, 2012; Z. Hu, Neural Development, 2011; M. K. Taylor, Development, 2007). We have cited these papers. Also, responding to this particular concern by the reviewer, we have also confirmed the expression of 3xHA_D1ICD_Flag in isolated NCSCs by FACS using anti-GFP antibody (Fig. S5B).

2.The authors claim activation of inducible mouse (CRE-ERT) based on tamoxifen injection. Moreover, they state that they can induce cre activity sparsely by decreasing tamoxifen concentration. The authors fail to demonstrate the cre efficiency within the specified area, or a larger fragment of the organism. A number of methods already available to the authors can directly address this concern.
We have cited the original paper describing UBC-CreERT2 transgenic line (Ruzankina et al., Cell Stem Cell. 2007 June 7; 1(1): 113-126.). We conducted the experiment according to their method and have also analyzed the recombinase efficiency of CreERT2 using GFP reporter line in each DRG (Fig.3). We additionally confirmed that the GFP expression was also sparsely observed in cortex in the embryos at E12.5 (below a picture). Green and blue represent GFP and DAPI, respectively.

We have removed unpublished data provided for the referees in confidence.

Accordingly, we have made the appropriate changes to the manuscript.
Before revision:
To ask the non-cell autonomous role of D1ICD in the neighboring cells, the protein was induced sparsely using a low dose of tamoxifen in the UBC-CreERT2/CAG-CAT-D1ICD mice.
After revision:
Thus, we attempted to induce D1ICD sparsely by tamoxifen injection in the UBC-CreERT2/CAG-CAT-D1ICD mice, then analyzed the role in the neighboring cells.

3.The authors do not mention or disclose control data (or its existence) in the biochemical experiments. The authors of this paper perform an immunoprecipitation of D1ICD and its interactors in HEK293T cells (human), because the pull down from their mutant mouse cells do not express enough of D1ICD (line 278-280;). However, in the previous fractionation experiment they determine the localization of substantial amounts of D1ICD in NCSCs (Figure S3G). The authors of the paper do not mention IgG control for the IP:D1ICD or an alternative control. Are the interactomes in the table EV1 representing all the proteins detected by mass spec? Are these listed proteins those that were specific for the IP:D1ICD as opposed to the IP:IgG control? This section is unclear.
To identify D1ICD-interacting proteins, HEK293T cells were transfected either with 3xFlag-tagged mouse D1ICD or empty vector, and immunoprecipitation was performed using anti-FLAG antibody. To omit nonspecific interactants, 1) we performed two independent experiments, 2) picked up proteins reproducibly identified from both of the two FLAG-D1ICD IPs, and 3) omitted proteins identified from one of the control IPs. The resultant list of interactants thus represents specific binders in table S1. The details are now shown in Table S2. The above-mentioned procedures have also been included in the methods section of the manuscript.

Furthermore, the authors do not show that they detect the D1ICD in the samples. This result questions the validity of their interactome, as the authors interpret their pulldown as successful based upon finding 2 proteins that have been previously been shown to interact with D1ICD.
We used human database for protein identification, as D1ICD (which derived from mouse sequence) was transfected into human HEK293T cells. To confirm successful precipitation of the bait, we also searched mouse database, and identified peptides corresponding to mouse D1ICD. The data are shown in Table S3 of the manuscript.

4.Finally, the title of the paper claims that they have revealed modulation of ErbB signaling, however the authors show only the changes in phosphorylated Erk1/2. The authors then state that pErk1/2 acts as an effector of Neuregulin-ErbB signaling. They claim (line 321-323) to SHOW a functional link between Notch-Delta and NRG1-ErbB signaling pathways. The assumption that pErk1/2 is, in this example, specific to ErbB signaling is overinterpreted because the Erk1/2 molecule is an effector in a number of different signaling pathways. No data on neuregulin has been shown in this manuscript.

© 2021. Published by The Company of Biologists under the terms of the Creative Commons Attribution License (https://creativecommons.org/licenses/by/4.0/).

We agree with the reviewer's criticism. We have eliminated the statements which mention that D1ICD modulates NRG-ErbB signaling in the title, introduction, result and discussion section.

Title:
Before revision:
Notch-reverse signaling regulates neural development via modulating both Notch and ErbB signalings
After revision:
Cleaved Delta like 1 intracellular domain regulates neural development via Notch signal dependent and independent pathways

Introduction:
Before revision:
Regarding the molecular mechanism, we found that D1ICD acts as a component of the lateral inhibition by up-regulating Numb expression, and connects the Notch and ErbB signals by repressing Notch activity and Erk1/2 phosphorylation.
After revision (Line 92-95):
With regards to the underlying molecular mechanism, we found that D1ICD acts as a component of the lateral inhibition by cooperating with Numb to repress Notch signaling, and that D1ICD also represses the MAP kinase pathway by inhibiting Erk1/2 phosphorylation.

Results:
Deletion:
It is reported that Erk1/2 act as effectors of Neuregulin1 (NRG1)-ErbB signaling which regulates peripheral nervous system development. Therefore, our results showed the functional link between Notch-Delta and NRG1-ErbB signaling pathways via D1ICD-mediated inhibition of ERK phosphorylation.

Discussion (Lines 367-385):
Changes made in the manuscript:
DRG glial cells consist of satellite glia and Schwann cells at E12.5 (Balakrishnan et al., 2016). It is well known that NRG1-ErbB signaling plays a crucial role in Schwann cell development (Jessen and Mirsky, 2019; Newbern and Birchmeier, 2010; Riethmacher et al., 1997). Moreover, NRG1 restricts NCSCs to a glial fate, and the isoform NRG1 type II promotes its differentiation into satellite glia, whereas the isoform NRG1 type III promotes its differentiation into Schwann cell (Hagedorn et al., 2000; Leimeroth et al., 2002; Shah et al., 1994). These results suggest that ErbB signaling promotes gliogenesis and cell survival during DRG development. Erk1/2 functions as effectors of ErbB signaling and play an important role in the survival of DRG-derived glial cells (Newbern et al., 2011). We found that D1ICD binds not only Erk1/2 but also Grb2, which is required for Erk1/2 activation mediated by ErbB signaling (Fig. 8D and Table 1) (Mei and Nave, 2014). These results indicate the possibility that D1ICD also regulates gliogenesis in coordination with ErbB signaling by inhibiting the Erk1/2 phosphorylation. In this study, however, we did not elucidate the direct interaction of D1ICD and Erk1/2 in DRG; thus, our results implicates an indirect consequence of changes in proliferation/differentiation. Thus, further analysis of interaction of DICD and ErbB signaling is an important aspect to be considered for future studies.
Collectively, we propose a model in which D1ICD plays a crucial role in DRG development via two mechanisms: the modulation of the lateral inhibition mechanism by inhibiting Notch signaling (Fig. S6B), and repression of the MAP kinase pathway by inhibitingErk1/2 phosphorylation (Fig. S6C).

Minor concerns:
1.Authors of this manuscript must enclose information as to whether the mutant mice are littermates of the corresponding controls. Since there are significant size differences in the tissues, it is important to know the relationship of the compared mice.
We conducted NC-Dll1 analysis using littermate embryos (Fig. 1I-L, Fig. 4, Fig. 7, Fig. 8H-J). We added experimental information analyzing littermate embryos in their figure legends.

© 2021. Published by The Company of Biologists under the terms of the Creative Commons Attribution License (https://creativecommons.org/licenses/by/4.0/).

2.In several areas the grammar needs to be corrected – sometimes entire fragments of the manuscript are extremely difficult to understand such as line 59, line 170, line 182, lines 189-197. In general many sections are excessively wordy with sentences that are too long.

We corrected the grammar in the following sections.
Line 59
Before revision:
In the developing mammalian nervous system, Delta-like 1 (Dll1), induced by the proneural genes Ascl1 and Neurog2 with in a cell, activates Notch signaling in the neighboring cells.
After revision:
In the developing mammalian nervous system, Notch which is expressed on signal-receiving cells, is activated by expression of Delta-like 1 (Dll1) in neighboring signal-sending cells.
Line 170: Deleted in the revised text.
Line 203.
Before revision:
To ask the non-cell autonomous role of D1ICD in the neighboring cells, the protein was induced sparsely using a low dose of tamoxifen in the UBC-CreERT2/CAG-CAT-D1ICD mice.
After revision:
Thus, we attempted to induce D1ICD expression sparsely by tamoxifen injection in the UBC-CreERT2/CAG-CAT-D1ICD mice, and analyzed its role in neighboring cells.
Line 189-197: Deleted in the revised text.

3.Authors should show that the fractionation experiment was successful by proper controls for each fraction (fig S3G), e.g. GAPDH, lamin B1, etc.

We performed western blotting using anti-Lamin B1 and GAPDH antibodies (Fig. S5C). Lamin B1 signal was only detected in the nuclear fraction. GAPDH expression was also detected in nuclear fraction, but the signal is weaker when compared with the cytoplasm fraction. It is reported that GAPDH is not restricted to the cytosol, it is also found in the nucleus although the expression in nucleus is lesser when compared to the expression found in the cytoplasm (Zhai et al. Molecular Brain 2014, 7:20). Accordingly, our results also showed that the nuclear fractionation experiment was successful.

4.Given the lack of controls in previous parts I would like to see controls showing that the Erk1/2 immunostaining (Fig 6D) is specific.

We showed the specificity of the pErk1/2 signal in the neural tube (below a picture). Green and red indicate pErk1/2 signal and NICD signal, respectively. We have also provided the antibody datasheet URL for the reviewer's perusal:
https://www.cellsignal.jp//products/primary-antibodies44-42-mapk-erk1-2-137f5-rabbit-mab/4695

© 2021. Published by The Company of Biologists under the terms of the Creative Commons Attribution License (https://creativecommons.org/licenses/by/4.0/).

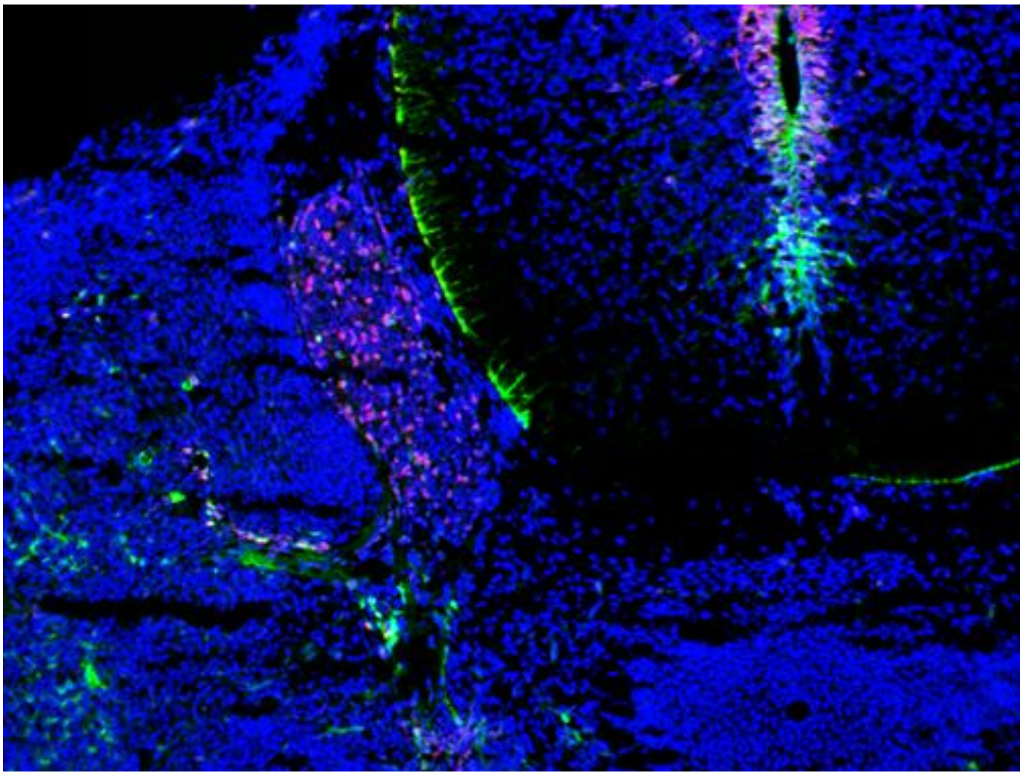

Reviewer #3:
This paper examines whether the intracellular domain (D1ICD) of the Notch ligand Delta1 regulates signalling and differentiation cell autonomously in addition to the more conventional function of Delta to signal through Notch to neighbouring cells. It has previously been shown that mice can develop apparently normally when engineered to suppress cleavage of Delta1. However this does not exclude the possibility that D1ICD influences development in relatively subtle ways that are not essential for viability.
Our study is the first one which is involved in investigating the suppression of Dll1 cleavage. There are a few reports which mention that D1ICD overexpression has little effects on mouse embryogenesis.

1) Does D1ICD really have an 'unconventional' autonomous intracellular role as the authors propose? An alternative possibility is that their experimental manipulations of Dll1 are interfering artificially with the conventional function of Dll1 to signal to neighbouring cells through Notch (already known to influence differentiation in the DRG). This would then indirectly result in changes to the Delta-manipulated cells due to Notch-mediated lateral inhibition.
The authors go some way towards supporting their intepretation (i.e a distinct cell-autonomous role for D1ICD) by showing that uncleavable Dll1 is able to activate Notch in NIH3T3 cells using a luciferase assay. However it seems important to provide evidence that "conventional" juxtacrine function of Dll1 is not artefactually compromised or over-stimulated when they suppress Dll1 cleavage *in DRGs*, particularly given their finding in Fig4 that D1ICD does actually seem to influence conventional Notch signalling in neighbouring cells.

Previously, we showed that there was no difference in Notch signal sending ability in NIH3T3 to neighboring cell between wildtype- and NC- Dll1, indicating that NC-Dll1 does not overstimulate Notch signaling (Fig. S2D). However, the NIH3T3 cell line may not be appropriate to represent DRG development. Thus, to reproduce DRG development as close to the *in vivo* conditions as possible, we investigated the expression of the Notch target gene *Hes1* and *Hey1* by transfecting wild-type- or NC- Dll1 into NCSC instead of NIH3T3. The expression of *Hes1* and *Hey1* was not altered by the overexpression of Dll1 or NC-Dll1, although there was some tendency for a decrease in the *Hes1* and an increase *Hey1* levels (Fig. S2E). There is a possibility that amount of transfected Dll1 and NC-Dll1 is not enough to affect for *Hes1* and *Hey1* expression. Thus, we analyzed the ratio Hes1/ Hey1 expression to the total Dll1

© 2021. Published by The Company of Biologists under the terms of the Creative Commons Attribution License (https://creativecommons.org/licenses/by/4.0/).

expression, our results showed that this ratio is significantly decreased in both transfected cells compared with control, but did not change between Dll1 and NC-Dll1, as shown in the below graph: Hes1 / Dll1, Hes1 / NC-Dll1, Hey1 / Dll1, Hey1 / NC-Dll1 were 0.019±0.005, 0.014±0.001, 0.031±0.007 and 0.023±0.001, respectively. These results indicate that the function of NC-Dll1 as a Notch ligand is not so different from the native Dll1 even in NCSC.

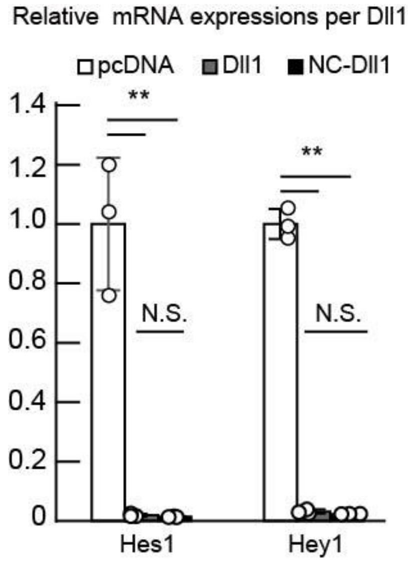

In addition, we have examined D1ICD function by *in vivo* and *in vitro* gain-of-function experiments. We found that overexpressed D1ICD promoted neuronal differentiation and inhibited Notch signaling in a cell autonomous manner (Fig. 2D-F, Fig. 3). D1ICD repressed Hey1 promoter activity induced by NICD in NIH3T3 cells cell-autonomously (Fig. 6C). D1ICD bound to Erk1/2 and subsequently inhibited its phosphorylation (Fig. 8 B-D). D1ICD also promoted neuronal differentiation in a Notch signal independent manner (Fig. 8 A). These results suggest that D1ICD has a biological function in DRG development.

In summary, the results obtained from using NC-Dll1 mice is not due to overstimulation of Notch signaling in adjacent cells, but rather to cell-autonomous repression of Notch signaling by suppression of D1ICD production.

2) Quantification of phenotypes
The authors document phenotypes by quantifying immunofluorescence in sections of DRGs. The message of the paper relies entirely on these quantifications because there is no reported functional defect in the mice (other than anecdotal mention of hyperactivity, data not shown). However there is no information on how quantification is performed- the materials and methods states that quantification was performed by counting cells in Photoshop rather than by using computational segmentation algorithms. It is not stated how many cells were counted from how many sections of how many DRGS, nor is it stated what criteria were used for scoring cells. Were sections scored blinded or not? I'm concerned that the methods used may be subject to inadvertent user bias. Given the relatively subtle nature of some of the phenotypes I think this raises a major concern about the reliability of the quantification on which the reported phenotypes are based. The authors could confirm their findings with an unbiased approach such as analysis of gene expression in acutely isolated cells from the DRG. They already describe experiments in which Delta-manipulated cells are sorted from the DRG and cultured in vitro for further phenotypic analysis, but it is important that they also analyse gene expression in *acutely* isolated cells in order to back up their immunofluorescence phenotyping data.
We have mentioned our cell counting method below.
We could not score as blind because the cell number of DRG was clearly distinguishable among wild-type, D1ICD overexpression driven by Wnt1Cre and NC-Dll1.

We did not use computational segmentation algorithms but counted signal positive cells using the following criteria. We distinguished each cell utilized DAPI staining. Thus, we could

© 2021. Published by The Company of Biologists under the terms of the Creative Commons Attribution License (https://creativecommons.org/licenses/by/4.0/).

discriminate the proteins localized in nucleus such as ki67, Sox10, NICD and pErk1/2. We also were able to clearly detect the proteins expressing a small population, such as cleaved caspase 3 and p75. GFP protein is recognized as a distinct single cell by the staining using anti-GFP antibody (ab13970, Abcam) and DAPI. Therefore, GFP reporter expressing cells by crossing with Wnt1Cre line or UBC-CreERT2 line can also be counted by using antibodies against each marker protein. Tuj1 and BFABP expression is found mainly in the cytoplasm. To clearly distinguish between cells expressing these proteins in wild-type and NC-Dll1 DRGs, only cells in which cytoplasmic staining clearly surrounded or covered the nucleus were counted as a single positive cell. Dll1 is also expressed predominantly in the cytoplasm. Thus, we determined the cell boundaries based on Dll1 staining of the cytoplasm and DAPI staining of the nuclei. We only counted a small number of Dll1 expressing cells in a small population of Notch signal active cells (Fig. S4D).

In addition to the above results, we investigated gene expression in acutely isolated DRG in wild-type and NC-Dll1 embryos as the reviewer has suggested. We have focused on thoracic DRG in the cell counting analysis. However, we analyzed pooled thoracic and lumber DRGs to obtain the requirement of total RNA quantity in the gene expression experiments. DRG development proceeds along the anteroposterior axis. It does indicate that the total RNA contains several developmental time points and thus, it concerns that the qPCR experiments are not sensitive compared with the cell counting method. In fact, the marker gene expressions such as Tuj1, BFABP, Hes1, Hey1 and Numb did not change significantly between wild-type and NC-Dll1 DRG because of a large standard deviation. Nevertheless, the ratio of Tuj1 per BFABP was decreased in NC-Dll1 DRG (Fig. 4F). In the second wave neurogenesis, common progenitors differentiate into neuronal or glial cells. The balance is regulated by Notch signaling via lateral inhibition mechanism (Hu et al., 2011; Taylor et al., 2007; Wakamatsu et al., 2000). These results suggest that the repression of D1ICD production promoted cell differentiation toward glial cells.

Moreover, we showed supportive data for the cell counting results.
1. D1ICD promoted neuronal differentiation of NCSCS in both Notch signaling active (Fig. S5A) and inactive (Fig. 8A) conditions; data corresponding to Fig. 2D-F, Fig. 3, Fig. 4A, B, E.
2. The phosphorylation of Erk1/2 also repressed in NCSCs (Fig. 8B &C) corresponding to Fig. 8E-G.

These results suggest that our quantification methods are appropriate.

3) Mechanism
It is unclear whether effects on Erk and Numb are direct consequence of interaction with Dl1 or an indirect consequence of changes to conventional Notch activity and subsequent changes to proliferation and differentiation.
I suggest that Erk activity and Numb expression should be measured in Dl1-manipulated cells isolated acutely from the DRG (rather than after a period of cell culture). Even if successful, this still leaves open the possibility of indirect effects and so this should be discussed as a caveat within the paper.
We have found *Numb* expression was increased in NCSC overexpressing D1ICD compared with control NCSC. However, we found *Numb* expression did not change significantly between acutely isolated wild-type and NC-Dll1 DRGs. Thus, we eliminated the description for D1ICD function increasing *Numb* mRNA expression.

In this study, we elucidated that D1ICD repressed Notch signaling in a cell autonomous manner through Numb/Numbl in NIH3T3 cell (Fig. 6). These results suggest that D1ICD plays a role in a component of lateral inhibition mechanism by inhibiting Notch signaling coordinated with Numb.

We have shown the direct interaction D1ICD and Erk1/2 in NCSC, but not in DRG. Thus, we have also mentioned in the manuscript that the results might indicate be an indirect consequence of changes in proliferation or differentiation, and that further analysis between DICD and MAP kinase pathway should be an important topic to be considered in the future studies.

© 2021. Published by The Company of Biologists under the terms of the Creative Commons Attribution License (https://creativecommons.org/licenses/by/4.0/).

Additional comments
Fig 2D/E please show TUJ1 staining alone in the DRG (i.e. not overlaid with GFP) to make it easier to compare whether or not there is really more TUJ1 in D1ICD DRGs. In the images shown the higher levels of GFP in controls tend to obscure the TUJ1 staining.
We added Tuj1 staining alone pictures in both control and D1ICD sustained expressed DRGs (Fig. 2D&E).

Fig 2 G-J Several markers are shown in G-J but it is difficult to see how many cells are positive for these markers because the large number of orange arrows obscure the image. Can the images be shown in a way that makes it easier for us to compare?
We added signal positive cell number in each picture (Fig. 3A-H).

Fig4 looks at cell autonomous vs. non autonomous effect using low-dose tamoxifen to induce DlCD in a few cells. There is some evidence that D1ICD cells are found next to cells with activated Notch, but we are not told how many cells are scored or how it is determined objectively whether a cell is a neighbour to a green cell. I found it difficult to understand how to reliably interpret these data.
We have listed out the number of counted cells below.
ERT2+D1ICD-: The number of active Notch signals in GFP negative cells surrounding the GFP positive cell, and total Notch active cells were 16, 24, 18 and 35, 57, 45, respectively.
ERT2+D1ICD+: The number of active Notch signals in GFP negative cells surrounding the GFP positive cell, and total Notch active cells were 16, 28, 29 and 27, 47, 54, respectively.
We determined a cell is a neighbor to a GFP positive cell by taking in regards the nuclei location.

Fig5: I found it difficult to understand the rationale for the experimental design here. If the claim is that Dl1ICD activates Numb why is there no analysis of Numb expression in vivo in D1ICD-overexpressing DRGS or uncleavable-D1 DRGS? Or in acutely isolated cells from those DRGs? We are only shown analysis of cells after isolation and culture.
D1ICD-overexpressing DRG driven by Wnt1Cre was too small to obtain enough quantity of total RNA. Thus, we change the interpretation.

5A: If the authors wish to convince us that their isolated DRG cells can differentiate into three lineages I'd suggest that they do more than just show us a single example of a cell from each lineage.
We eliminated Fig 5A and cited proper reference (Nagoshi, N *et al.*, et al. 2008, Cell Stem Cell 2, 392-403.).

5D shows an experiment in 3T3 cells that seems to be designed to test whether Numb is required for Dl1ICD to influence Notch target genes based on a luciferase assay. I found this impossible to understand- the graph is not labelled and not explained in the legend.
We apologize the incomplete figure. We have re-written the figure legend.

Fig6: The authors use an assay based on 'isolated' single cells to exclude the possibility that Dl1ICD is influencing juxtacrine signalling in this experimental setup. I found this unconvincing- neural progenitors should divide and generate neighbours for themselves in culture, and if they do not then they are probably either damaged cells or differentiated cells rather than neural progenitors. Furthermore, it is not stated how many cells were scored and how many cells were excluded from this assay.
To dispel reviewer's concern, we have eliminated the result and conducted new NCSC differentiation assay withdrawal growth factors for five days treated with g-secretase inhibitor compound E to repress endogenous Notch signaling. D1ICD also promoted neuronal differentiation even when the endogenous Notch signaling is repressed (Fig. 8A).

Fig6: The authors should discuss the possibility that the observed differences in pErk may be an indirect consequence of changes in proliferation/differentiation rather than an upstream cause of these changes.
We have added the below description in Discussion section of the manuscript (Line 362-366).
Erk1/2 bind to D1ICD in NCSC, thereby suggesting that the MAP kinase pathway is a direct target of D1ICD. However, we did not elucidate the direct interaction of D1ICD and Erk1/2 in DRG,

© 2021. Published by The Company of Biologists under the terms of the Creative Commons Attribution License (https://creativecommons.org/licenses/by/4.0/).

thus the results might indicate be an indirect consequence of changes in proliferation or differentiation. Further analysis between DICD and Map kinase pathway should be an important subject in the future studies.

We very much hope that the additional experiments we performed and changes to the text we have made to this revised version of our manuscript are satisfactory and that you and your referees are now happy to publish this study in *Development*.

Second decision letter

MS ID#: DEVELOP/2020/193664

MS TITLE: Cleaved Delta like 1 intracellular domain regulates neural development via Notch signal dependent and independent pathways

AUTHORS: Yusuke Okubo, Fumiaki Ohtake, Katsuhide Igarashi, Yukuto Yasuhiko, Yoko Hirabayashi, Yumiko Saga, and Jun Kanno

I have now received the reports of the three referees who reviewed the earlier version of your manuscript and I have reached a decision. The referees' comments are appended below, or you can access them online: please go to BenchPress and click on the 'Manuscripts with Decisions' queue in the Author Area.

The overall evaluation is positive and we would like to publish a revised manuscript in Development, provided that you satisfactorily address the remaining suggestions and comments of referee 3. This referee asks in particular that you provide information throughout the paper on the numbers of cell analysed, and from how many sections, how many DRGs, etc. The referee also asks that you soften your claim that Delta-IC acts cell autonomously, present it as a suggestion and discuss also the possibility that the phenotypes you see may be due to non-cell-autonomous. disruption of conventional Notch signalling. Please attend to all these comments in your revised manuscript and detail them in your point-by-point response. If you do not agree with any of their criticisms or suggestions, explain clearly why this is so.

We are aware that you may currently be unable to access the lab to undertake experimental revisions. If it would be helpful, we encourage you to contact us to discuss your revision in greater detail. Please send us a point-by-point response indicating where you are able to address concerns raised (either experimentally or by changes to the text) and where you will not be able to do so within the normal timeframe of a revision. We will then provide further guidance. Please also note that we are happy to extend revision timeframes as necessary.

Reviewer 1

Advance summary and potential significance to field

The authors sought to elucidate the function of the intracellular domain of Delta-like1 (D1ICD) in the developing dorsal root ganglia (DRG) by generating Dll1-overexpressing and NC-Dll1 mice. Interestingly D1ICD overexpression inhibited cell proliferation and accelerated neurogenesis whereas inhibition of D1ICD formation (NC-Dll1) decreased neurogenesis and increased the DRG cell number. D1ICD inhibited Notch signaling partly by inducing Numb, which could act as a Notch inhibitor. Furthermore, D1ICD inhibited phosphorylation of Erk1/2 by physical interaction and thereby promoted neuronal differentiation. This is an interesting work showing the in vitro and in vivo functions of D1ICD.

© 2021. Published by The Company of Biologists under the terms of the Creative Commons Attribution License (https://creativecommons.org/licenses/by/4.0/).

Comments for the author

The authors properly answered my comments, and I have no further comments.

Reviewer 2

Advance summary and potential significance to field

The authors have shown that the D1ICD plays a role in stem cell fate decision during development using in vivo and in vitro approaches. Authors have made significant contribution to understanding of role of Delta outside of its heavily studied role in Notch activation. The manuscript will be of broad interest to both developmental and stem cell biology communities and is appropriate for Development.

Comments for the author

The authors have completed most suggested experiments, revised their manuscript and edited the interpretations to successfully improve the quality of the data and manuscript for publication in Development. Without reservation, I now recommend it for publication.

Reviewer 3

Advance summary and potential significance to field

See previous review

Comments for the author

The authors have clearly tried hard to address my concerns. They have performed a number of additional experiments and clarified their description of some of their analysis methods. Unfortunately, the new data and information they provide does not go very far to alleviate my concerns. Looking at the bigger picture, however, I recognise that the paper reports a clear phenotype from their two mouse models, based on DRG size/proliferation, and that this supports their overall claim that “Cleaved Delta like 1 intracellular domain regulates neural development”. The paper is therefore of some interest to readers of Development . However the reliability of some of the evidence remains in doubt and the insights into the underlying mechanism remain relatively weak. It might be that these points can be dealt with by softening claims and explaining caveats.

I’ve listed my main concerns below.
1) Original concern re. overall model
I wrote: “Does D1ICD really have an 'unconventional' autonomous intracellular role as the authors propose? An alternative possibility is that their experimental manipulations of Dll1 are interfering artificially with the conventional function of Dll1 to signal to neighbouring cells through Notch”
The authors originally used NIH 3T3 cells to show that their uncleavable Delta is not compromised in its ability to activate Notch. I asked them to provide similar evidence in DRGs but they were unable to provide such evidence due to technical problems. The new data in FigS2E does not show any response to either cleavable or uncleavable Delta. The data provided in the rebuttal on this point is in my view invalid: rather than showing changes in expression of a Notch target gene (e.g. Hes1) in response to Delta vs uncleavable Delta, they show instead the *ratio* of Hes1 to Delta. Given that Delta is overexpressed in this experiment then this ratio is of course massively reduced for both variants of Delta compared to controls. In my view this gives no meaningful information about Notch response and I’m not sure why the authors have included this graph in an attempt to support their case.

They also make the point: “D1ICD promoted neuronal differentiation and inhibited Notch signalling in a cell autonomous manner (Fig. 2D-F, Fig. 3)” but as discussed below these data seem to be based on quantification of very small numbers of cells and therefore don’t seem to be particularly

© 2021. Published by The Company of Biologists under the terms of the Creative Commons Attribution License (https://creativecommons.org/licenses/by/4.0/).

reliable (but I'm happy to be corrected on this point if it is a misunderstanding, as discussed below).

This concern also relates to Fig5, which provides evidence that DeltaIC inhibits Notch cell-autonomously and activates Notch in neighbouring cells. Why do they propose that this is explained by a novel mechanism (DeltaIC is directly inhibiting Notch cell autonomously) rather than by the well-established consequence of conventional lateral inhibition (Delta activates Notch in neighbouring cells, and this results in downregulation of Notch ligands and consequently a loss of Notch activity in the original Delta-expressing cell)?
Overall, my concern on this point still stands. If the paper is published then in my view the authors should soften their claims that Delta-IC acts cell autonomously and they should discuss the possibility that the phenotypes they see may be due to non-cell-autonomous. disruption of conventional Notch signalling.

2) Original concern re quantification:
I wrote: "It is not stated how many cells were counted from how many sections of how many DRGS, nor is it stated what criteria were used for scoring cells. Were sections scored blinded or not? I'm concerned that the methods used may be subject to inadvertent user bias. Given the relatively subtle nature of some of the phenotypes I think this raises a major concern about the reliability of the quantification on which the reported phenotypes are based."
The authors give a reasonable explanation for not scoring blind (because the size of the DRG reveals the genotype).They explain that they scored cells by eye rather than using computational image analysis- this is not unreasonable, although it does reduce confidence in the data. However, importantly they still do not state how many cells were counted from how many sections of how many DRGs in Figure 2 and in several other places in the paper (unless I missed this somewhere?). It is not clear if they scored entire DRGs or only the cells within the box shown on the figures. This means I'm still uncertain whether the quantification of IF shown throughout the paper is acceptable. My concern therefore still stands unless the authors can correct me on this point. Given my uncertainty over quantification of IF I suggested in my original report: "The authors could confirm their findings with an unbiased approach such as analysis of gene expression in acutely isolated cells from the DRG." The authors attempted this experiment but were unable to convincingly confirm any reproducible and significant changes in expression of Tuj1, BFABP, Hes1, Hey1 or Numb, and although they did see a modest change in the ratio Tuj1 /BFABP this falls quite a long way short of confirming the findings of their IF quantification. I accept that this lack of confirmation can be explained by technical difficulties and should not be taken as evidence against their model, and I appreciate the efforts they have made to address this point,. Nevertheless, unfortunately this does not go very far towards bolstering confidence in their claims.
I suggest that the authors either reassure us that they scored a reasonable number of cells in each experiment and provide this information within the paper, or alternatively remove any quantification where this reassurance cannot be given (noting that this will reduce the strength of evidence quite considerably).

3) Original concern re: whether effects on Erk and Numb are relatively direct or indirect.
My original report said: "I suggest that Erk activity and Numb expression should be measured in Dl1-manipulated cells isolated acutely from the DRG (rather than after a period of cell culture)"
I thank the authors for doing this experiment. Unfortunately they found that Numb was not affected in acutely isolated cells so they have removed this claim from their paper (they have however left in their claim that DeltaIC affects Numb in 3T3 cells (Fig 6)).
The authors have not been able to demonstrate a direct interaction between N1ICD and Erk1/3 in DRGs, but this could be for technical reasons rather than because an interaction does not exist, and they do provide some data from NCSC
Overall, the authors have not been able to address my concern on this point despite making a good effort to do so. They have therefore modified their text to allow for the possibility of indirect rather than direct effects on Numb/Erk, which is fine but does mean that the paper still lacks solid insights into the mechanism by which Delta-IC operates. Perhaps they should soften their statements about mechanism given that their proposed mechanisms remain somewhat speculative.

4) Original concern re quantification of non-autonomous effects:
I wrote: "Fig4 looks at cell autonomous vs. non autonomous effect using low-dose tamoxifen to induce DlCD in a few cells. There is some evidence that D1ICD cells are found next to cells with

© 2021. Published by The Company of Biologists under the terms of the Creative Commons Attribution License (https://creativecommons.org/licenses/by/4.0/).

activated Notch, but we are not told how many cells are scored or how it is determined objectively whether a cell is a neighbour to a green cell. I found it difficult to understand how to reliably interpret these data."
I'm afraid I don't understand the authors response on this point, and I see no change in the figure or main text to clarify this point for the readers of the paper. From what I can gather, only small numbers of cells are scored. Perhaps the authors can clarify further, but for now I have to say that they do not seem to have addressed my concern on this point.

5) Original concern re original Fig6, which aimed to show that DeltaIC is influencing juxtracrine Notch signalling.
The authors agree with my criticism of their experimental design and have removed the original data and provided an entirely new experiment based on using inhibitors of endogenous Notch activity (new Fig 8). This is an interesting new way to address this point, but I can't see how figure 8A supports their claims. First, if the Notch inhibitor is working properly should it not increase neurogenesis also in the absence of exogenous D1ICD? Second, they state "D1ICD also promoted neuronal differentiation even when the endogenous Notch signalling is repressed". Where does the word "also" come in here? The data seem to show that D1ICD does NOT increase TUJ1 expression in the absence of the Notch inhibitor, which doesn't seem to be in keeping with their overall model. Perhaps I'm misunderstanding this figure and the authors could explain more clearly how it supports their point.

Second revision

Author response to reviewers' comments

TITLE: Cleaved Delta like 1 intracellular domain regulates neural development via Notch signal dependent and independent pathways
AUTHORS: Yusuke Okubo, Fumiaki Ohtake, Katsuhide Igarashi, Yukuto Yasuhiko, Yoko Hirabayashi, Yumiko Saga, and Jun Kanno

Dear. Dr. Francois Guillemot
Editor, *Development*

We are grateful for the reviewer's constructive suggestions, which led us to perform these additional experiments and make the appropriate changes to the manuscript, which we feel have significantly improved the paper. The text below provides our detailed response, highlighted in bold, following each suggestion/comment of the corresponding reviewer. We have also highlighted these revisions in the revised manuscript.

1 : This referee asks in particular that you provide information throughout the paper on the numbers of cell analysed, and from how many sections, how many DRGs, etc.

We have added the number of scoring cells throughout the paper in Table S4. As mentioned by Reviewer 3, it seems to be a small number of counting cells; however, we performed supporting experiments for the scoring. The results shown in Fig. S5A, Fig. 4F, and Fig. 8B support the results corresponding to the enhancement of neuronal differentiation by D1ICD overexpression (Fig. 2D-F, Fig. 3), inhibition of neuronal differentiation by repression of D1ICD production (Fig. 4A-E), and the inhibition of Erk1/2 phosphorylation (Fig. 8E, F).

2 : The referee also asks that you soften your claim that Delta-IC acts cell autonomously, present it as a suggestion and discuss also the possibility that the phenotypes you see may be due to non-cell-autonomous disruption of conventional Notch signalling.

We understand Reviewer 3's criticism of our claim that D1ICD acts in cell autonomously. We have removed the description of D1ICD working in cell autonomously and added that D1ICD affects conventional Notch-Delta signaling in the Discussion section.

© 2021. Published by The Company of Biologists under the terms of the Creative Commons Attribution License (https://creativecommons.org/licenses/by/4.0/).

Reviewer 3 Comments for the Author:
The authors have clearly tried hard to address my concerns. They have performed a number of additional experiments and clarified their description of some of their analysis methods. Unfortunately, the new data and information they provide does not go very far to alleviate my concerns. Looking at the bigger picture, however, I recognise that the paper reports a clear phenotype from their two mouse models, based on DRG size/proliferation, and that this supports their overall claim that “Cleaved Delta like 1 intracellular domain regulates neural development”. The paper is therefore of some interest to readers of Development . However the reliability of some of the evidence remains in doubt and the insights into the underlying mechanism remain relatively weak. It might be that these points can be dealt with by softening claims and explaining caveats.

In response to Reviewer 3's request, we have removed the description against D1ICD works in cell autonomously and added the descriptions mentioning that D1ICD may act by affecting conventional Notch-Delta signaling in the Discussion section. In addition, we have added the scoring cell number in Table S4 and the details of the cell counting method in the Supplemental Methods.

We have also changed the following descriptions.
Abstract:
Line: 39
Before revision:
D1ICD acts as an integral component of lateral inhibition by the inhibition of Notch activity in a cell-autonomous manner.
After revision:
D1ICD acts as an integral component of lateral inhibition mechanism by inhibiting Notch activity.

Results:
Line: 188
Before revision:
D1ICD acts cell-autonomously to repress the Notch activity and non-cell autonomously to activate adjacent cells via lateral inhibition mechanism.
After revision:
D1ICD acts as an integral component of the lateral inhibition mechanism by the repressing Notch activity.

Line: 200
Before revision:
We analyzed the cell-autonomous effects by inducing D1ICD in mice embryo by crossing the Wnt1-Cre with the CAG-CAT-D1ICD mice.
After revision:
To determine whether D1ICD participates in Notch signaling pathway, we analyzed Notch activity-induced embryos produced by crossing the Wnt1-Cre with the CAG-CAT-D1ICD mice.

Line: 204
Before revision:
Moreover, the D1ICD overproduction enhanced BFABP expression in the neighboring cells (Fig. S3B, C), implying that D1ICD could also control the cell fate in a non-cell-autonomous manner.

After revision:
Moreover, the D1ICD overproduction enhanced BFABP expression in the neighboring cells (Fig. S3B, C).

Line: 240
Before revision:
We next investigated the Notch activity in the NC-Dll1 DRG.
After revision:
To further investigate the possible role of D1ICD, we examined the Notch activity in NC-Dll1 DRG that lacks D1ICD production.

© 2021. Published by The Company of Biologists under the terms of the Creative Commons Attribution License (https://creativecommons.org/licenses/by/4.0/).

Line: 242
Before revision:
We also found that the ratio of cells co-stained with the NICD and Dll1 C-terminal epitope in NICD-positive cells did not change significantly between wild-type and NC-Dll1 DRGs (Fig. S4 D, E). The ratio of the co-stained cells in total cells was significantly increased in NC-Dll1 compared to wild-type DRGs (Fig. S4 F). These results suggest that the repression of Dll1 expression by Notch signaling through the lateral repression mechanism is also appropriate for NC-Dll1 DRGs. Moreover, overproduction of D1ICD repressed Notch activity in a cell-autonomous manner (Fig. 5C, D). Thus, the result could be interpreted that the inhibition of D1ICD production in the NC-Dll1 DRG increased the Notch activity cell-autonomously (Fig. S4G). Taken together, these results indicate that D1ICD works as a component of lateral inhibition mechanism via repressing the Notch activity in a cell-autonomous manner.
After revision:
Notch activity is regulated via a lateral inhibition mechanism. In our experiment, Notch activity and Dll1 expression showed a roughly mutually exclusive pattern in the wild-type DRG at E12.5 (Fig. 1A). Therefore, we explored whether the up-regulation of Notch signaling in NC-Dll1 DRG was caused by the disruption of the lateral inhibition mechanism. We found that Notch activity and Dll1 expression showed a salt-and-pepper pattern in NC-Dll1 as shown in the wild-type, and the ratio of cells co-stained with the NICD and Dll1 C-terminal epitope in NICD-positive cells did not change significantly between the wild-type and NC-Dll1 DRGs (Fig. S4D, E). These results indicate that the lateral inhibition mechanism also worked properly even in NC-Dll1, and the up-regulation of Notch activity is because of a lack of Notch activity modulation by D1ICD rather than disruption of the lateral inhibition mechanism. Taken together, these results suggest that D1ICD functions as a component of the lateral inhibition mechanism by repressing Notch signaling in own cell.

Line: 268
Before revision:
D1ICD significantly increased Tuj1 expression in Notch signal-inhibited NCSCs (Fig. 8A).
After revision:
We confirmed that Notch signaling was inhibited by Compound E treatment because *Hes1* mRNA expression was decreased (Fig. S5B). D1ICD significantly increased Tuj1 expression even in the NCSCs treated with Compound E (Fig. 8A), although under these experimental conditions, we did not observe up-regulation of Tuj1 only by D1ICD (see discussion).

Line: 313
Before revision:
Linkage between Notch signaling and MAP kinase activity via D1ICD-mediated Erk inhibition.
After revision:
D1ICD suppressed phosphorylation of Erk 1/2

Line: 318
Before revision:
These results indicate that D1ICD binds to Erk1/2 and inhibits their phosphorylation in a cell-autonomous manner during the second wave neurogenesis. As described above, D1ICD represses Notch signaling in cells-autonomous manner and activates Notch signaling in the neighboring cells (Fig. 5A-D).

After revision:
These results indicate that D1ICD binds to Erk1/2 and inhibits their phosphorylation during the second wave neurogenesis. As described above, D1ICD represses Notch signaling in own cells and activates Notch signaling in the neighboring cells (Fig. 5A-D).

Discussion:
Line: 331
Before revision:
Second, D1ICD works as a component of lateral inhibition mechanism by inhibiting Notch activity in coordination with Numb. Thirdly, D1ICD binds Erk1/2, and inhibits their phosphorylation in a

© 2021. Published by The Company of Biologists under the terms of the Creative Commons Attribution License (https://creativecommons.org/licenses/by/4.0/).

cell-autonomous manner. Finally, the MAP kinase pathway and the Notch signaling linked via D1ICD.

After revision:

Second, D1ICD functions as an integral component of the lateral inhibition mechanism by suppressing Notch signaling. Third, D1ICD represses Notch activity in coordination with Numb in NIH3T3 cells. Finally, D1ICD binds Erk1/2 and inhibits their phosphorylation in a cell-autonomous manner.

We added the below descriptions.
Results:
Line: 214

Thus, D1ICD expressing cells might repress their own Notch activity.

Discussion:
Line: 359

Furthermore, our study revealed that D1ICD promotes neuronal differentiation in NCSCs in a Notch signal-independent manner (Fig. 8A). As shown in Fig. S5A, we found that D1ICD-overexpressing NCSCs significantly increased Tuj1 in the differentiation assay upon withdrawal of growth factors. In contrast, D1ICD did not increase Tuj1 expression (Fig. 8A), although we used the same NCSC population as the material. The only difference was the absence (Fig. S5A) or presence (Fig. 8A) of DMSO. It has been reported that the mRNA expression of the neuronal marker *Doublecortin* decreased in adult rat neural stem and precursor cells treated with 1% DMSO, indicating that low concentrations of DMSO suppress neuronal differentiation (O'Sullivan et al., 2019). Therefore, we speculated that low concentrations of DMSO also inhibited neuronal differentiation in the NCSCs. Nevertheless, based on the strong up-regulation of Tuj1 by D1ICD even in the presence of Compound E, we conclude that D1ICD promotes neuronal differentiation even when endogenous Notch signaling is repressed. Moreover, D1ICD inhibited the phosphorylation of Erk1/2 during the second wave neurogenesis (Fig. 8B-I).

Line: 381

In this study, although we could not address the role of endogenous D1ICD in Dll1 function, which activates Notch signaling in neighboring cells, the intracellular domain of Notch ligands is shown to be ubiquitinated for their processing, which is required for the maintenance and activation of Notch signaling in neighboring cells (Dutta et al., 2021). Thus, D1ICD may regulate Notch signaling not only in a cell-autonomous manner but also in a non-cell-autonomous manner.

Methods:
Line: 459

Each score was calculated by counting each marker-positive cell of the whole DRG in one section from one embryo. The number of counted cells is shown in Table S4. The details of the quantification are described in Supplemental Methods.

We removed the below descriptions.
Previous line 201:

Since D1ICD is produced from Dll1, D1ICD primarily works as a cell-autonomous factor, however, whether D1ICD also participates in the Notch signaling pathway that influences the fate of neighboring cells, remained to be answered.

Previous line: 218

These results imply that D1ICD inhibits the Notch activity cell-autonomously, which may lead to an increase in the expression of Dll1, thereby activating Notch signal in the neighboring cells.

Previous line252:

Moreover, overproduction of D1ICD repressed Notch activity in a cell-autonomous manner (Fig. 5C, D). Thus, the result could be interpreted that the inhibition of D1ICD production in the NC-Dll1 DRG increased the Notch activity cell-autonomously (Fig. S4G).

Previous line362:

© 2021. Published by The Company of Biologists under the terms of the Creative Commons Attribution License (https://creativecommons.org/licenses/by/4.0/).

Our study revealed that D1ICD inhibits the phosphorylation of Erk1/2 during the second wave neurogenesis in a cell-autonomous manner, whereas it promotes the Erk1/2 phosphorylation in a non-cell-autonomous manner. Erk1/2 bind to D1ICD in NCSC, thereby suggesting that the MAP kinase pathway is a direct target of D1ICD.

Previous line 369:

DRG glial cells consist of satellite glia and Schwann cells at E12.5 (Balakrishnan et al., 2016). It is well known that NRG1-ErbB signaling plays a crucial role in Schwann cell development (Jessen and Mirsky, 2019; Newbern and Birchmeier, 2010; Riethmacher et al., 1997). Moreover, NRG1 restricts NCSCs to a glial fate, and the isoform NRG1 type II promotes its differentiation into satellite glia, whereas the isoform NRG1 type III promotes its differentiation into Schwann cell (Hagedorn et al., 2000; Leimeroth et al., 2002; Shah et al., 1994). These results suggest that ErbB signaling promotes gliogenesis and cell survival during DRG development.

I've listed my main concerns below.
1) Original concern re. overall model

I wrote: "Does D1ICD really have an 'unconventional' autonomous intracellular role as the authors propose? An alternative possibility is that their experimental manipulations of Dll1 are interfering artificially with the conventional function of Dll1 to signal to neighbouring cells through Notch"

The authors originally used NIH 3T3 cells to show that their uncleavable Delta is not compromised in its ability to activate Notch. I asked them to provide similar evidence in DRGs but they were unable to provide such evidence due to technical problems. The new data in FigS2E does not show any response to either cleavable or uncleavable Delta. The data provided in the rebuttal on this point is in my view invalid: rather than showing changes in expression of a Notch target gene (e.g. Hes1) in response to Delta vs uncleavable Delta, they show instead the *ratio* of Hes1 to Delta. Given that Delta is overexpressed in this experiment then this ratio is of course massively reduced for both variants of Delta compared to controls. In my view this gives no meaningful information about Notch response and I'm not sure why the authors have included this graph in an attempt to support their case. They also make the point: "D1ICD promoted neuronal differentiation and inhibited Notch signalling in a cell autonomous manner (Fig. 2D-F, Fig. 3)" but as discussed below these data seem to be based on quantification of very small numbers of cells and therefore don't seem to be particularly reliable (but I'm happy to be corrected on this point if it is a misunderstanding, as discussed below). This concern also relates to Fig5, which provides evidence that DeltaIC inhibits Notch cell-autonomously and activates Notch in neighbouring cells. Why do they propose that this is explained by a novel mechanism (DeltaIC is directly inhibiting Notch cell autonomously) rather than by the well-established consequence of conventional lateral inhibition (Delta activates Notch in neighbouring cells, and this results in downregulation of Notch ligands and consequently a loss of Notch activity in the original Delta-expressing cell)?
Overall, my concern on this point still stands. If the paper is published then in my view the authors should soften their claims that Delta-IC acts cell autonomously and they should discuss the possibility that the phenotypes they see may be due to non-cell-autonomous. disruption of conventional Notch signalling.

We believe that the results of D1ICD overexpression in most cells of the DRG using Wnt1Cre (Fig. 5A-D) cannot be explained by CONVENTIONAL Notch-Delta signaling. Reviewer 3 argues that D1ICD acts only on Notch signaling in neighboring cells via Dll1 of its own cells. If so, Notch activity should also be increased in D1ICD overexpressing cells by Dll1 in the surrounding abundant D1ICD overexpressing cells because endogenous Dll1 is expressed in most of the D1ICD-expressing cells (98.0 ± 3.4%) that constitute the majority of the DRG. In the DRG, the ratio of NICD-positive cells in the total cells was increased compared to the control (left graph below). However, while Notch is activated in GFP-negative cells that do not express D1ICD, Notch signaling is barely activated in GFP-positive cells that overexpress D1ICD (Fig. 5A, B and right graph below). In addition, we showed that D1ICD suppresses cell-autonomously transfected NICD-dependent Hey1 promoter activity in NIH3T3 cells (Fig. 6). Although Reviewer 3 has raised doubts about the immunostaining scoring method, as shown in Fig. 2E and F, almost all D1ICD-expressing cells (93.9 ± 2.5) were differentiated into neurons compared to 66.4% Tuj1-positive GFP-positive cells in the control. The difference

© 2021. Published by The Company of Biologists under the terms of the Creative Commons Attribution License (https://creativecommons.org/licenses/by/4.0/).

from the control was obvious (p = 0.0003045). This is supported by an *in vitro* experiment showing that neuronal differentiation is enhanced by D1ICD using NCSCs (Fig. S5A). Thus, we hypothesized that D1ICD represses Notch signaling in a cell-autonomous manner.

However, we could not show any difference in the signal sending ability between cleavable Dll1 and non-cleavable Dll1 in "DRG" as described by Reviewer 3 because of technical problems. This means that we cannot completely rule out the possibility that D1ICD may affect conventional Notch-Delta signaling. Therefore, we deleted or altered the description of cell-autonomous effects and discussed their effects on conventional Notch-Delta signals.

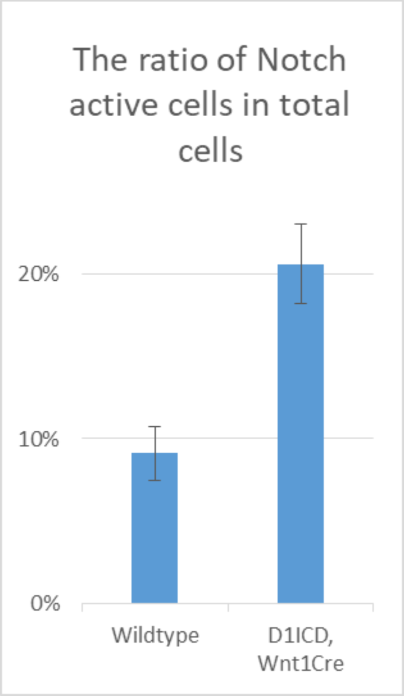

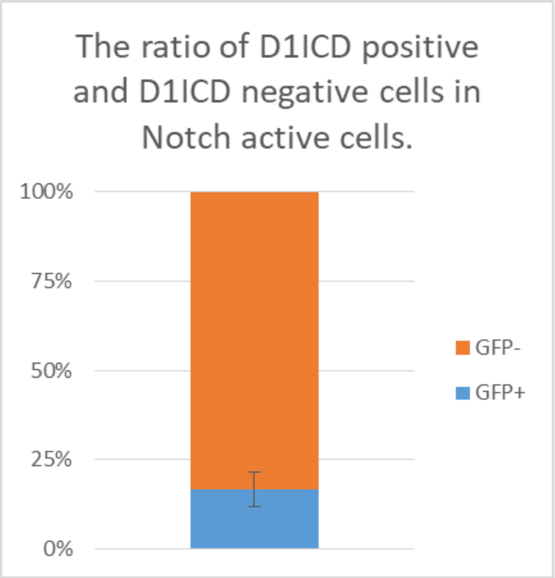

2)Original concern re quantification:

I wrote: “It is not stated how many cells were counted from how many sections of how many DRGS, nor is it stated what criteria were used for scoring cells. Were sections scored blinded or not? I'm concerned that the methods used may be subject to inadvertent user bias. Given the relatively subtle nature of some of the phenotypes I think this raises a major concern about the reliability of the quantification on which the reported phenotypes are based.”
The authors give a reasonable explanation for not scoring blind (because the size of the DRG reveals the genotype).They explain that they scored cells by eye rather than using computational image analysis- this is not unreasonable, although it does reduce confidence in the data. However, importantly they still do not state how many cells were counted from how many sections of how many DRGs in Figure 2 and in several other places in the paper (unless I missed this somewhere?). It is not clear if they scored entire DRGs or only the cells within the box shown on the figures. This means I’m still uncertain whether the quantification of IF shown throughout the paper is acceptable. My concern therefore still stands unless the authors can correct me on this point.

Given my uncertainty over quantification of IF I suggested in my original report: “The authors could confirm their findings with an unbiased approach such as analysis of gene expression in acutely isolated cells from the DRG.” The authors attempted this experiment but were unable to convincingly confirm any reproducible and significant changes in expression of Tuj1, BFABP, Hes1, Hey1 or Numb, and although they did see a modest change in the ratio Tuj1 /BFABP this falls quite a long way short of confirming the findings of their IF quantification. I accept that this lack of confirmation can be explained by technical difficulties and should not be taken as evidence against their model, and I appreciate the efforts they have made to address this point,. Nevertheless,

© 2021. Published by The Company of Biologists under the terms of the Creative Commons Attribution License (https://creativecommons.org/licenses/by/4.0/).

unfortunately this does not go very far towards bolstering confidence in their claims.

I suggest that the authors either reassure us that they scored a reasonable number of cells in each experiment and provide this information within the paper, or alternatively remove any quantification where this reassurance cannot be given (noting that this will reduce the strength of evidence quite considerably).

The scoring data are presented in Table S4. We have also added the following description of the scoring method: Each score was calculated by counting each marker-positive cell of the whole DRG in one section from one embryo.

Although the number of cells counted might be small, we have shown other experimental results that support our claim. The enhancement of neuronal differentiation by D1ICD overexpression *in vivo* was reproduced by a differentiation assay using NCSCs (Fig. S5A). In addition, we have shown that D1ICD promotes neuronal differentiation even in the presence of endogenous Notch signaling suppression, which is technically challenging to achieve *in vivo* (Fig. 8A). The results of the suppression of D1ICD production using NC-Dll1 embryos were also analyzed using marker mRNA expression of isolated DRGs proposed by Reviewer 3. Although the results were less sensitive because of technical limitations for reasons previously mentioned (and re-described below), the results revealed that the direction of DRG differentiation was towards glial differentiation (Fig. 4F). Furthermore, the suppression of Notch signaling in D1ICD overexpressing cells using NIH3T3 cells showed that D1ICD suppresses Notch signaling in a cell-autonomous manner (Fig. 6). However, we could not analyze the effect of D1ICD suppression on conventional Notch signaling in DRGs, as claimed by Reviewer 3, due to technical difficulties. Therefore, we added the description in the Discussion section that D1ICD may act by affecting conventional Notch-Delta signaling. In addition, we used NCSCs to determine the binding of D1ICD to Erk1/2 (Fig. 8D) and the inhibition of its phosphorylation (Fig. 8 C, D). These results are consistent with our quantification results.

Previous revision comment:
We analyzed pooled thoracic and lumbar DRGs to determine the requirement of total RNA quantity in the gene expression experiments. DRG development proceeded along the anteroposterior axis. This indicates that the total RNA contains several developmental time points, and thus, qPCR experiments are not sensitive compared with the cell counting method. Nevertheless, the ratio of Tuj1 to BFABP was decreased in the NC-Dll1 DRG (Fig. 4F). In the second wave neurogenesis, common progenitors differentiate into neuronal or glial cells. The balance is regulated by Notch signaling via a lateral inhibition mechanism (Hu et al., 2011; Taylor et al., 2007; Wakamatsu et al., 2000). These results suggest that repression of D1ICD production promotes cell differentiation toward glial cells.

3)Original concern re: whether effects on Erk and Numb are relatively direct or indirect.

My original report said: “I suggest that Erk activity and Numb expression should be measured in Dl1-manipulated cells isolated acutely from the DRG (rather than after a period of cell culture)”

I thank the authors for doing this experiment. Unfortunately they found that Numb was not affected in acutely isolated cells so they have removed this claim from their paper (they have however left in their claim that DeltaIC affects Numb in 3T3 cells (Fig 6)). The authors have not been able to demonstrate a direct interaction between N1ICD and Erk1/3 in DRGs, but this could be for technical reasons rather than because an interaction does not exist, and they do provide some data from NCSC. Overall, the authors have not been able to address my concern on this point despite making a good effort to do so. They have therefore modified their text to allow for the possibility of indirect rather than direct effects on Numb/Erk, which is fine but does mean that the paper still lacks solid insights into the mechanism by which Delta-IC operates. Perhaps they should soften their statements about mechanism given that their proposed mechanisms remain somewhat speculative.

© 2021. Published by The Company of Biologists under the terms of the Creative Commons Attribution License (https://creativecommons.org/licenses/by/4.0/).

We have shown that Notch signaling is repressed in D1ICD-expressing cells induced by Wnt1Cre. Therefore, we investigated the relationship between the D1ICD and Numb. In our previous revision, we found that D1ICD did not affect Numb expression. In contrast, we have shown that D1ICD cells autonomously repress NICD-inducible Hey1 promoter activity via Numb/Numbl function (Fig. 6). However, we have not been able to elucidate the cell-autonomous action of D1ICD in "DRGs," and therefore, we have altered the description of the cell-autonomous repression of Notch signaling by D1ICD. In accordance with the criticism of Reviewer 3, we have deleted the section on cooperation between Notch-Delta signaling and ErbB signaling via D1ICD from the Discussion section.

4) Original concern re quantification of non-autonomous effects:

I wrote: “Fig4 looks at cell autonomous vs. non autonomous effect using low-dose tamoxifen to induce DlCD in a few cells. There is some evidence that D1ICD cells are found next to cells with activated Notch, but we are not told how many cells are scored or how it is determined objectively whether a cell is a neighbour to a green cell. I found it difficult to understand how to reliably interpret these data.”

I’m afraid I don’t understand the authors response on this point, and I see no change in the figure or main text to clarify this point for the readers of the paper. From what I can gather, only small numbers of cells are scored. Perhaps the authors can clarify further, but for now I have to say that they do not seem to have addressed my concern on this point.

We added all counting cell numbers in Table S4.
GFP-negative cells surrounding GFP-positive cells were discriminated using GFP staining. Because whole GFP-positive cells were distinguishable as single cells, GFP-negative cells surrounding GFP-positive cells were distinguished using DAPI staining surrounding GFP-positive cells within a distance of 1.5-fold diameter of the nucleus in the GFP-positive cells from the outline of GFP-positive cells.
In addition to Fig. 5E and F, Notch signaling was active in cells neighboring D1ICD overexpressing cells induced by the Wnt1Cre line (Fig. 5A, B). These results suggest that D1ICD activates Notch signaling in neighboring cells, although additional experiments are needed to reveal the underlying mechanism.
We changed the image in Fig. 5E to discriminate GFP negative cells beside GFP expressing cells.

We added the following text into new Supplemental methods:
Signal-positive cells were counted using the following criteria: Each cell was stained with DAPI. The proteins localized in the nucleus, such as Ki67, Sox10, NICD, and pErk1/2, were counted only by co-staining with DAPI. Proteins expressing a small population, such as cleaved caspase 3 and p75, were clearly discriminated by the cell. GFP protein is recognized as a distinct single cell by staining with anti-GFP antibody (ab13970, Abcam) and DAPI. Therefore, GFP reporter-expressing cells crossing the Wnt1Cre line or UBC-CreERT2 line can also be counted using antibodies against each marker protein. Tuj1 and BFABP expression were found mainly in the cytoplasm. To clearly distinguish between cells expressing these proteins in wild-type and NC-Dll1 DRGs, only cells in which cytoplasmic staining clearly surrounded or covered the nucleus were counted as a single positive cell. Dll1 was also expressed predominantly in the cytoplasm. The cell boundaries were determined by Dll1 staining of the cytoplasm and DAPI staining of the nuclei. GFP-negative cells surrounding GFP-positive cells were discriminated using GFP staining. Because whole GFP-positive cells were distinguishable as single cells, GFP-negative cells surrounding GFP-positive cells were distinguished using DAPI staining surrounding GFP-positive cells within a distance of 1.5-fold diameter of the nucleus in the GFP-positive cells from the outline of GFP-positive cells.

5)Original concern re original Fig6, which aimed to show that DeltaIC is influencing juxtracrine Notch signalling.
The authors agree with my criticism of their experimental design and have removed the original data and provided an entirely new experiment based on using inhibitors of endogenous Notch activity (new Fig 8). This is an interesting new way to address this point, but I can’t see how figure

© 2021. Published by The Company of Biologists under the terms of the Creative Commons Attribution License (https://creativecommons.org/licenses/by/4.0/).

8A supports their claims. First, if the Notch inhibitor is working properly should it not increase neurogenesis also in the absence of exogenous D1ICD?

In addition to the new Fig. 8, we confirmed that *Hes1* expression was decreased in the NCSCs treated with compound E, indicating that Notch signaling was suppressed (new Fig. S5B). Furthermore, we re-examined the differentiation assay of the NCSCs and found that the expression of GFAP was suppressed by compound E treatment (new Fig. 8A). We also confirmed the Reviewer's comment that inhibition of Notch signaling promotes neuronal differentiation (new Fig. 8A).

Second, they state "D1ICD also promoted neuronal differentiation even when the endogenous Notch signalling is repressed". Where does the word "also" come in here? The data seem to show that D1ICD does NOT increase TUJ1 expression in the absence of the Notch inhibitor, which doesn't seem to be in keeping with their overall model. Perhaps I'm misunderstanding this figure and the authors could explain more clearly how it supports their point.

As shown in Fig. S5A, we found that D1ICD-overexpressing NCSCs significantly increased Tuj1 in the differentiation assay with only withdrawal growth factors. In contrast, D1ICD did not increase Tuj1 expression (Fig. 8A), although we used the same NCSC population as the material. The only difference was the absence (Fig. S5A) or presence (Fig. 8A) of DMSO. It has been reported that neuronal marker DCX mRNA expression was decreased in adult rat neural stem and precursor cells treated with 1% DMSO, indicating that low concentrations of DMSO suppress neuronal differentiation (ref.1). Therefore, we speculate that low concentrations of DMSO also inhibit neuronal differentiation in NCSCs. Nevertheless, based on the strong upregulation of Tuj1 by D1ICD even in the presence of Compound E, we conclude that D1ICD promotes neuronal differentiation even when endogenous Notch signaling is repressed.

Ref.1: Anna O'Sullivan et al., (2019). Dimethylsulfoxide Inhibits Oligodendrocyte Fate Choice of Adult Neural Stem and Progenitor Cells. *Frontiers in Neuroscience* 13, 1242.

Third decision letter

MS ID#: DEVELOP/2020/193664

MS TITLE: Cleaved Delta like 1 intracellular domain regulates neural development via Notch signal dependent and independent pathways

AUTHORS: Yusuke Okubo, Fumiaki Ohtake, Katsuhide Igarashi, Yukuto Yasuhiko, Yoko Hirabayashi, Yumiko Saga, and Jun Kanno
ARTICLE TYPE: Research Article

I am delighted to tell you that your manuscript has been accepted for publication in Development, pending our standard ethics checks.

© 2021. Published by The Company of Biologists under the terms of the Creative Commons Attribution License (https://creativecommons.org/licenses/by/4.0/).
